# Characterization of gene cluster heterogeneity in single-cell transcriptomic data within and across cancer types

**DOI:** 10.1242/bio.059256

**Published:** 2022-06-23

**Authors:** Khong-Loon Tiong, Yu-Wei Lin, Chen-Hsiang Yeang

**Affiliations:** 1 Institute of Statistical Science, Academia Sinica, 128 Academia Road, Section 2, Taipei 115, Taiwan; 2 The University of Texas MD Anderson Cancer Center, School of Health Profession, Master Program of Diagnostic Genetics, Houston, Texas, 77030, USA

**Keywords:** Single-cell RNA-sequencing, Tumor heterogeneity, Clustering, Pan-cancer analysis, Bulk level RNA-sequencing

## Abstract

Despite the remarkable progress in probing tumor transcriptomic heterogeneity by single-cell RNA sequencing (sc-RNAseq) data, several gaps exist in prior studies. Tumor heterogeneity is frequently mentioned but not quantified. Clustering analyses typically target cells rather than genes, and differential levels of transcriptomic heterogeneity of gene clusters are not characterized. Relations between gene clusters inferred from multiple datasets remain less explored. We provided a series of quantitative methods to analyze cancer sc-RNAseq data. First, we proposed two quantitative measures to assess intra-tumoral heterogeneity/homogeneity. Second, we established a hierarchy of gene clusters from sc-RNAseq data, devised an algorithm to reduce the gene cluster hierarchy to a compact structure, and characterized the gene clusters with functional enrichment and heterogeneity. Third, we developed an algorithm to align the gene cluster hierarchies from multiple datasets to a small number of meta gene clusters. By applying these methods to nine cancer sc-RNAseq datasets, we discovered that cancer cell transcriptomes were more homogeneous within tumors than the accompanying normal cells. Furthermore, many gene clusters from the nine datasets were aligned to two large meta gene clusters, which had high and low heterogeneity and were enriched with distinct functions. Finally, we found the homogeneous meta gene cluster retained stronger expression coherence and associations with survival times in bulk level RNAseq data than the heterogeneous meta gene cluster, yet the combinatorial expression patterns of breast cancer subtypes in bulk level data were not preserved in single-cell data. The inference outcomes derived from nine cancer sc-RNAseq datasets provide insights about the contributing factors for transcriptomic heterogeneity of cancer cells and complex relations between bulk level and single-cell RNAseq data. They demonstrate the utility of our methods to enable a comprehensive characterization of co-expressed gene clusters in a wide range of sc-RNAseq data in cancers and beyond.

## Abstract (precis)

**Summary:** We propose quantitative methods to analyze cancer sc-RNAseq data: measures of intra-tumoral heterogeneity, characterization of a hierarchy of gene clusters, and alignment of gene cluster hierarchies from multiple datasets.

## INTRODUCTION

Tumor heterogeneity is intensively investigated due to strong implications in understanding and tackling cancers such as reconstructing the evolutionary history of tumor subclones (e.g. Gerlinger et al., 2012; Wang et al., 2014), predicting the efficacy of immunotherapy (e.g. Kim et al., 2020; Jang et al., 2020; Lu et al., 2019; Gurjao et al., 2019), identifying the molecular basis of drug resistance (e.g. Kim et al., 2015; Suzuki et al., 2015), and adjusting regimen to cope with it (e.g. Beckman et al., 2012; Akhmetzhanov et al., 2019). Single-cell sequencing technologies are powerful tools to unravel tumor heterogeneity. DNA sequencing is the most mature technology among the single-cell measurements and has been extensively employed to detect subclonal structures of tumors (e.g. see a review by Tsoucas and Yuan, 2017). Single-cell RNA sequencing (abbreviated as sc-RNAseq) was developed later but has been widely used to investigate both intra-tumoral and inter-tumoral transcriptomic heterogeneity pertaining to more transient processes.

Sc-RNAseq data has demonstrated diversity and complexity of cell types (cancer cells, immune cells and stromal cells) within tumors (Zhao et al., 2018; Darmanis et al., 2017; Nguyen et al., 2016; Zhang et al., 2020). It has been employed to chart the tumor immune microenvironment (e.g. Kim et al., 2020, Close et al., 2020; Jang et al., 2020, and Lu et al., 2019) and the stromal cell microenvironment (e.g. Lambrechts et al., 2018, Peng et al., 2019), cluster cancer cells or identify new cell types according to their expression profiles (e.g. Peng et al., 2019; Zhang et al., 2019; Yue et al., 2020; Wu et al., 2018; Young et al., 2018; Horning et al., 2018), acquire dynamic information such as origins, evolution and development of tumor subclones (e.g. Peired et al., 2020; Praktiknjo et al., 2020; Kester and van Oudenaarden, 2018; Foerink et al., 2018; Teschendorff and Enver, 2017; Park et al., 2016; Lei et al., 2016), presence of cancer stem cells or quantification of cancer stemness (e.g. Wu et al., 2019; Peixoto et al., 2019, Chen et al., 2019). Inter-tumoral heterogeneity is often reported from bulk level sequencing data (e.g. Hoadley et al., 2018). Investigations using sc-RNAseq data make additional contributions by comparing the subtype compositions of tumors with distinct pathological types, clinical traits and treatment responses (e.g. Zheng et al., 2018; Kashima et al., 2018; Davis et al., 2020) and identifying differentially expressed genes between distinct groups of tumors (e.g. Vinogradov and Anatskaya, 2020; Zhu et al., 2018; Li et al., 2018, Zhang et al., 2018, 2016, Freeman et al., 2016).

Despite the remarkable progress in investigating tumor heterogeneity with single-cell sequencing technologies, several gaps exist in the bioinformatics analyses of cancer sc-RNAseq data. First, the term tumor heterogeneity appears frequently in the literature of cancer genomics but is not formally defined or quantified. Second, although clustering analyses have been widely employed in sc-RNAseq data, most of them target cells rather than genes. Furthermore, gene clusters are typically characterized by functional enrichment but not differential levels of transcriptomic heterogeneity. Third, relations between gene clusters inferred from multiple datasets/cancer types remain less explored. Alignment of gene clusters across datasets is useful for pan-cancer data analysis but not well developed.

To fill those gaps, we provided a series of quantitative methods to cluster genes in cancer sc-RNAseq data, quantify their heterogeneity, and align the gene clusters across multiple datasets. They consist of three major components. First, we proposed two quantitative measures – NSV and *p*_*diff*_ scores – based on correlation coefficients of expression profiles to assess intra-tumoral heterogeneity/homogeneity of the whole transcriptomes or gene clusters. Second, we applied a consensus k-means clustering algorithm to establish a hierarchy of gene clusters with varying *k*’s, devised an algorithm to reduce the gene cluster hierarchy to a compact structure, and characterized the gene clusters with functional enrichment and heterogeneity. Third, we developed an algorithm to align the gene cluster hierarchies from multiple datasets and generate a small number of meta gene clusters.

We applied these algorithms to simulated data and nine sc-RNAseq datasets covering seven cancer types. Both NSV and *p*_*diff*_ scores recovered known intra-tumoral heterogeneity in simulated data and outperformed the widely used entropy metric. For each sc-RNAseq dataset, we discovered that cancer cell transcriptomes were more homogeneous within tumors than the accompanying normal cells. Gene clusters in each dataset possessed distinct levels of heterogeneity and functional enrichment. Many gene clusters across nine datasets were aligned to two largest meta gene clusters, which had high and low intra-tumoral heterogeneity and were enriched with cell cycle/DNA repair and cellular respiration/antigen processing and presentation, respectively. Finally, we investigated the relations between the cluster structures derived from single-cell and bulk level RNAseq data by verifying the gene clusters from one dataset to another. This rich information justifies the utility of our sc-RNAseq analysis algorithms.

## RESULTS

### Two quantitative measures capture intra-tumoral heterogeneity

We propose a collection of tumors are heterogeneous if cells within tumors are more dissimilar to cells between tumors. This notion differs from the classical definition of population diversity (such as Simpson's index, Simpson, 1949 or effective number of species, Chao et al., 2016), which concerns the number of subtypes (species) and their composition in tumors (communities). We argue our notion is more adequate for tumor sc-RNAseq data since it can capture the relational properties of multiple tumors rather than the population compositions of individual tumors and does not require subjective demarcation of subtypes based on transcriptomes.

We propose two quantitative measures to assess intra-tumoral heterogeneity of sc-RNAseq data. The first measure is based on silhouette values in clustering analysis (Rousseeuw, 1987). Data points are the expression values of cells over selected genes or the whole transcriptome, and distances between data points are defined in a Euclidean space. Euclidean distances are sensitive to the scale of expression data in each cell. To mitigate this problem we normalized the expression vector of each cell to a z-score with zero mean and unit variance, so that and the Euclidean distance between the two z-score vectors was 2−2*ρ*, where *ρ* was the correlation coefficient between the two expression vectors.

A cluster of data points corresponds to the cells belonging to the same tumor. Silhouette values quantify the relative strength of intra-cluster (intra-tumoral) to inter-cluster (inter-tumoral) distances. More precisely, for a cell with index *i*, denote ***x***_*i*_ its normalized expression vector (z-score), *l*_*i*_ the index of the tumor it belongs to, and *a*(*i*) the average intra-tumoral distance of cell *i*: 
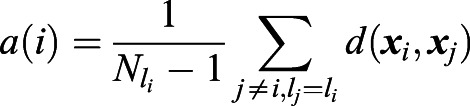
, where 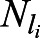 is the number of cells with tumor label *l*_*i*_. Likewise, denote *b*(*i*) the minimum average inter-tumoral distance of cell *i*: 
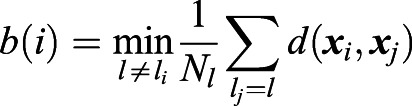
. The silhouette value is defined as:
(1)

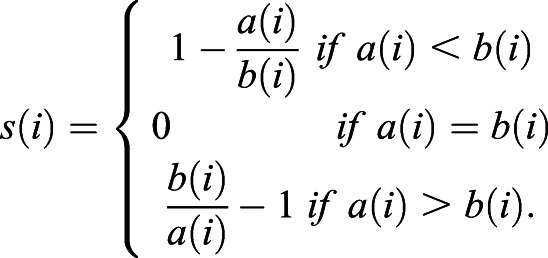
From this definition − 1≤*s*(*i*)≤1. *s*(*i*)<0 if cell *i* is more similar to the cells in another tumor than to the cells in its own tumor (*a*(*i*)>*b*(*i*)). Therefore, by our definition a tumor is heterogeneous if it contains a high proportion of cells with negative silhouette values. For each tumor (patient) we counted the fraction of cells with negative silhouette values (NSV). The average NSV fraction over all tumors therefore indicates the level of intra-tumoral heterogeneity.

The second measure of intra-tumoral heterogeneity is to compare the distributions of correlation coefficients between cells within and across tumors. We computed the correlation coefficients of expression data (over selected genes or the whole transcriptomes) between all pairs of cells and subdivided them into intra-tumoral (cell pairs from the same tumors) and inter-tumoral (cell pairs from distinct tumors) groups. Tumors are less heterogeneous (or more homogeneous) if intra-tumoral correlation coefficients are generally higher than inter-tumoral correlation coefficients. Standard statistical tests quantifying deviation between non-parametric distributions (such as Kolmogorov-Smirnov and Mann-Whitney tests) are very sensitive to sample sizes. In our case, intra and inter tumoral correlation coefficients constitute hundreds or thousands of cells and thus tens of thousands or millions of pairs. A tiny deviation between the two distributions with such large sample sizes will yield a drastically small *P*-value. To diminish sensitivity to sample sizes, we proposed an alternative statistical test to quantify deviation between distributions. Denote *p*_*intra*_ and *p*_*inter*_ the intra-tumoral and inter-tumoral correlation coefficient distributions. *p*_*intra*_ and *p*_*inter*_ can be viewed as the underlying distributions of two random variables *X* and *Y*, respectively. We quantified the deviation from *p*_*intra*_ to *p*_*inter*_by a measure *p*_*diff*_≡*P*(*X*>(*Y*+*ε*))−*P*(*X*<(*Y*−*ε*)), where *ε* is a small number (0.05 in this study). To evaluate *p*_*diff*_, we generated a large number of *X* and *Y* instances by rejection sampling from *p*_*intra*_ and *p*_*inter*_ and calculated *P*(*X*>(*Y*+*ε*)) and *P*(*X*<(*Y*−*ε*)) from sampled data.

We demonstrated the adequacy of these two indices to capture intra-tumoral heterogeneity or homogeneity in simulated data. The toy expression data comprised ten genes and 500 cells sampled from five populations (100 cells per population). In each population, the expression vector of each cell was a constant vector plus a zero-mean, *σ*^2^-variance Gaussian noise. The 500 cells were allocated to five tumors in three cases: case 1 – each tumor contained 100 cells exclusively from one population, case 2 – each tumor contained 60 cells from one population and ten cells from each of the remaining four populations, case 3 – each tumor contained 20 cells from each population. Besides NSV and *p*_*diff*_ scores, we also calculated the average Shannon's entropy over tumors for the three cases.

Table S1 reports the three indices of the three cases with varying *σ*’s of the Gaussian noise. Cases 1-3 have increasing levels of intra-tumoral heterogeneity (or decreasing levels of intra-tumoral homogeneity). This trend is captured by all three indices at all noise levels (increasing levels of NSV and entropy and decreasing levels of *p*_*diff*_). However, while the gaps of entropy scores considerably reduce with increasing noise levels (the gap score between case 3 and case 1 is 8.00 for *σ*=0.05 and 1.79 for *σ*=0.5), the gaps of NSV and *p*_*diff*_ scores are much more robust (the gap scores are 0.716 and 0.498 for NSV and 1.009 and 0.499 for *p*_*diff*_, respectively). Furthermore, calculating entropy in a high-dimensional data is generally intractable due to the difficulty of joint density function estimation, unless simplifying assumptions are imposed (for instance, we assumed expressions of each gene were independent).

### Transcriptomes of cancer cells possess more intra-tumoral heterogeneity than the normal cell counterparts

We collected and processed nine datasets of single-cell cancer transcriptomes whose summary information is listed in Table 1. Six of those datasets consist of both cancer and normal cells (primarily immune cells). To better understand the origin of transcriptomic heterogeneity in cancer cells, we compared intra-tumoral homogeneity of cancer and normal cells by projecting the transcriptomic data of individual cells onto a two-dimensional plane using T-distributed Stochastic Embedding (t-SNE) (van der Maaten and Hinton, 2008) and visually inspecting their distributions within and across patients, and computing the NSV and *p*_*diff*_ scores of cancer and normal cells in each dataset.

**Table 1.**
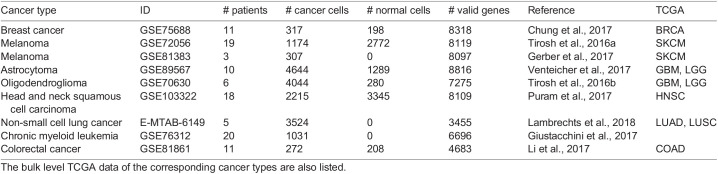
Summary information of the nine sc-RNAseq datasets in the present study

**Fig. 1.**
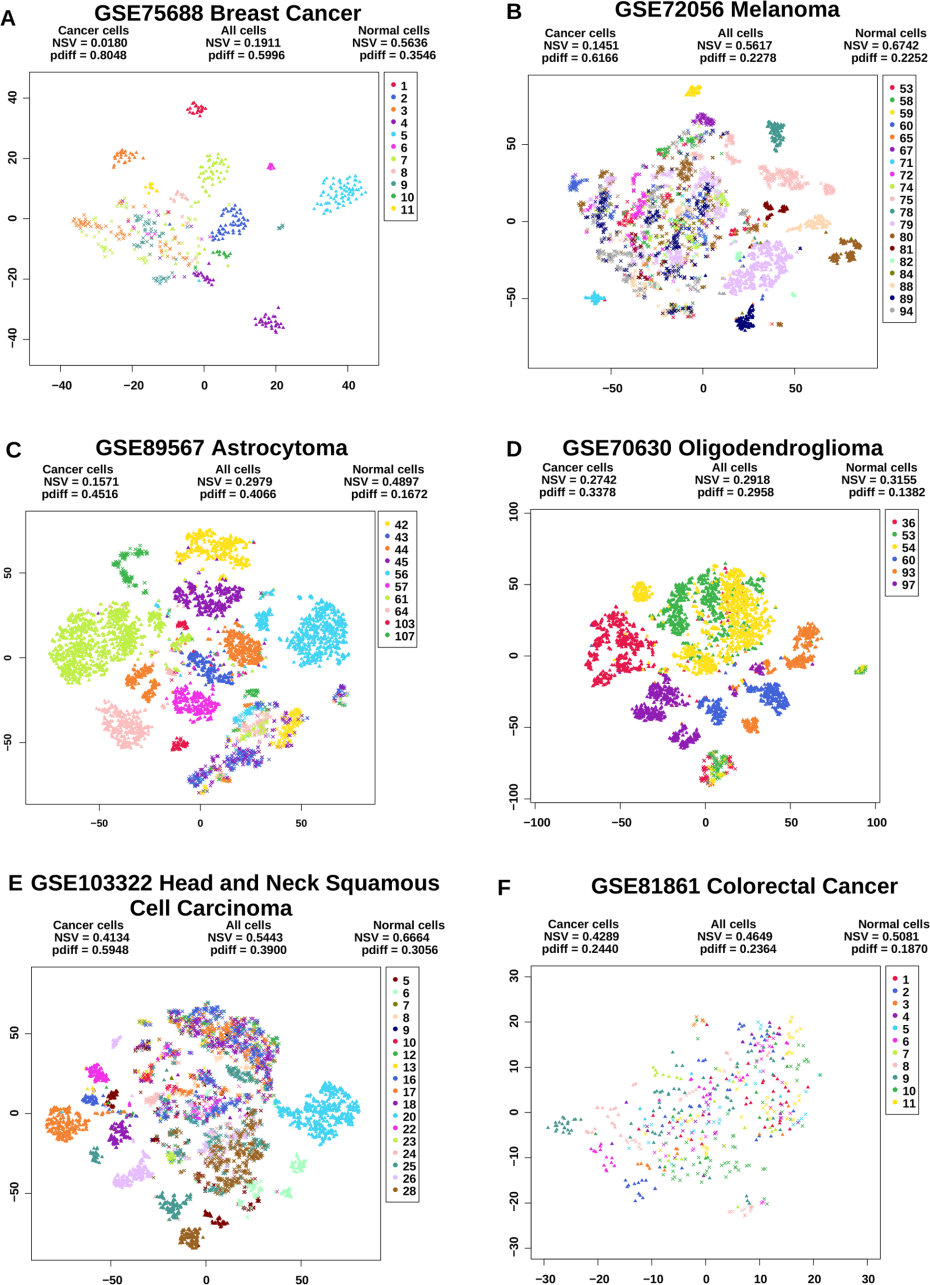
**t-SNE plots of transcriptomes of normal and cancer cells in six sc-RNAseq datasets.** Triangles and crosses denote cancer and normal cells respectively. Cells from distinct tumors (patients) are colored differently. The two heterogeneity scores – average NSV fractions and *p*_*diff*_ – of all cells, normal and cancer cells of each dataset are reported.

Fig. 1 displays the t-SNE plots of cancer and normal cells together from six datasets and reports their average NSV fractions and *p*_*diff*_ scores. In all but one dataset (GSE81861 colorectal cancer), cancer cells (triangles) are widely separated by their tumor (patient) identities. In contrast, normal cells (crosses) are separated primarily by their types but are intermingled between tumors. Visual inspection is compatible with the two quantitative measures as cancer cells by and large have lower average NSV fractions and higher *p*_*diff*_ scores than the normal cell counterparts.

High levels of intra-tumoral heterogeneity in normal cells are primarily confounded by the diverse cell types captured in the data. To account for this confounder, we visualized the t-SNE plots and reported the average NSV fractions and *p*_*diff*_ scores of individual cell types separately (Figs S1–S6). For instance, in GSE75688 breast cancer data cancer cells have larger *p*_*diff*_ (0.8048) and smaller NSV (0.0180) than normal cells aggregated (0.5636 and 0.3546, respectively, Fig. 1A). Intra-tumoral heterogeneity in normal cells reduces considerably after stratification by cell types, but still exceeds intra-tumoral heterogeneity in cancer cells. In GSE75688 data, cancer cells still have larger *p*_*diff*_ and smaller NSV than those in each of the four normal cell types separately (Fig. S1). Therefore, transcriptomes of cancer cells are more homogeneous within tumors than each type of normal cells.

### Gene clusters emerged from distinct cancer types exhibit differential levels of intra-tumoral heterogeneity and functional enrichment

Transcriptomic homogeneity of cancer cells displayed in Fig. 1 and Figs S1–S6 is attributed to the expression data of many genes. To deconstruct transcriptomic heterogeneity, we clustered genes according to their cancer cell expressions in each dataset, assessed intra-tumoral heterogeneity and functional enrichment of each gene cluster, and examined relations between enriched functions and heterogeneity.

We clustered genes using a consensus k-means clustering (Wilkerson and Hayes, 2010). The number of clusters (*k*) is a free parameter and often determined by context-specific means. As an illustrative example we first set *k*=7 and examined several features associated with each gene cluster – intra-tumoral homogeneity (*p*_*diff*_) and density (fraction of non-dropout entries in the data), false discovery rate (FDR) adjusted enrichment *P*-values (Benjamini and Hochberg, 1995) of 5745 gene sets (Subramanian et al., 2005), and enrichment *P*-values of marker gene groups reported in the papers of the six datasets. Later we varied *k* from 1 to 12 and represented clustering outcomes as a hierarchy.

The gene set enrichment *P*-values and summary information of the examined features are reported in Tables S2A and S2B, respectively. The enriched gene sets are either universally and highly enriched in the majority of datasets (type 1, *P*-values ≤ 10^−6^ in at least six datasets), or moderately enriched in specific datasets (type 2, *P*-values ≤ 10^−3^ in one or two datasets). Type 1 gene sets comprise roughly four large functional categories: ribosome, respiration, RNA splicing and cell cycle. Cell cycle and RNA splicing gene sets are co-enriched in the same clusters of some datasets, while other functional categories are enriched in separate clusters of most datasets. The *p*_*diff*_ (homogeneity) scores of the gene clusters harboring these functional categories generally follow an order of ribosome>respiration>RNA splicing>cell cycle. Type 2 gene sets are mostly related to the functions pertaining to the tissues of origins for the cancer types, such as pigmentation in melanoma datasets, neuron projection and differentiation in brain tumor datasets, hematopoiesis and leukocyte differentiation in CML dataset. Most marker gene groups are co-enriched with some gene sets in the same clusters. Instances include HER2+ markers and interferon gamma response in GSE75688 breast cancer data (cluster 2), and SOM D markers and epithelial-mesenchymal transition in GSE81383 melanoma data (cluster 3).

The *p*_*diff*_ scores and densities of gene clusters are moderately correlated (correlation coefficient 0.539). Clusters with low densities tend to have low *p*_*diff*_ scores and are enriched with cell cycle, and clusters with high densities tend to have high *p*_*diff*_ scores and are enriched with ribosome.

To ensure the features of intra-tumoral heterogeneity, density and functional enrichment are robust against the number of clusters, we varied *k* from 1 to 12, reported stable clusters for each *k*, and devised a compact representation for the clustering outcomes. Clusters between consecutive levels (*k*’s) possess overlap relations. A cluster at level *k*+1 may be split from one cluster at level *k* or joined by subsets of genes from multiple clusters at level *k*. We define an inheritance relation between clusters at consecutive levels. Cluster *c*_*j*_ at level *k*+1 inherits cluster *c*_*i*_ at level *k* if their overlap size ≥ 15% of the size of *c*_*j*_. The resulting clusters and their inheritance relations constitute a hierarchy or directed acyclic graph. This hierarchy differs from the dendrogram obtained from hierarchical clustering as one cluster may possess multiple parents (if it is joined with multiple clusters at the higher level) or multiple children (if it splits into multiple clusters at the lower level). A full hierarchy is not a compact representation since it may consist of clusters that remain highly overlapped over a range of *k*'s. We term these clusters a *stump* as they constitute a single branch (one child for each parent) over multiple levels in the hierarchy. To reduce the full hierarchy into a compact representation, we developed an algorithm to detect all stumps and collapse each stump into a node. In brief, it identifies the maximal paths where all the non-terminal nodes have single parents and children (stumps), collapses stumps into nodes, and reassigns parents and children of the reduced nodes. A detailed description of cluster hierarchy reduction algorithm is reported in the Materials and Methods.

The inheritance relations of clusters in the hierarchy also make their functional enrichment outcomes correlated. For instance, if cluster *c*_1_ splits to clusters *c*_2_ and *c*_3_, *c*_1_ and *c*_2_ are both significantly enriched with a GO term *g* but *c*_3_ is not, then it is inadequate to assign function *g* to *c*_1_ since members of *g* are likely concentrated in one sublineage *c*_2_ of *c*_1_. In contrast, if *g* is enriched in all three clusters, then it is adequate to assign *g* to *c*_1_ since members of *g* are likely distributed in both sublineages of *c*_1_. A functional category is defined as uniquely enriched in a gene cluster if its enrichment cannot be attributed to the enrichment concentrated in any substructure of the gene cluster. We proposed an algorithm to infer uniquely enriched gene clusters of a functional category. In brief, it identifies the maximal path in the hierarchy encompassing the top-ranking gene clusters in terms of enrichment *P*-values. The bottom node along this path is reported as the uniquely enriched gene cluster. A detailed description of this algorithm is reported in the Materials and Methods.

**Fig. 2.**
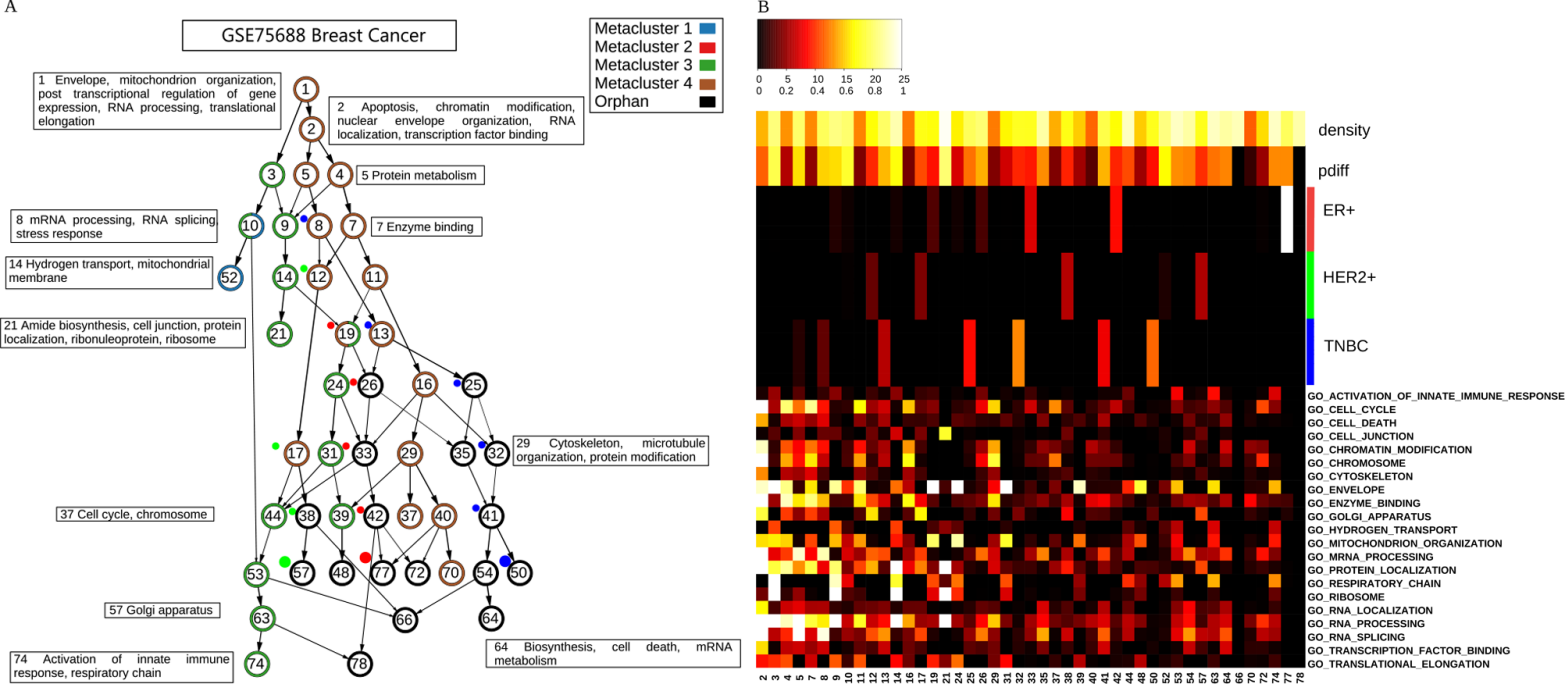
**Summary information of clustering results of GSE75688 breast cancer data.** The left hierarchy (A) displays the reduced hierarchy derived from stable k-means clusters. A node denotes a gene cluster. An edge denotes an inheritance relation from a higher-level cluster (a cluster generated by a larger *k*) to a lower-level cluster (a cluster generated by a smaller *k*). The thickness of an edge reflects the overlap level (intersection size over the higher-level cluster size). Uniquely enriched selected GO terms of clusters are annotated in boxes. The meta gene cluster identities of gene clusters are annotated by the circumference colors of nodes. Some gene clusters belong to multiple meta gene clusters, and the orphan clusters without meta gene cluster assignments are colored by black. The colored dots along some lineages denote significant enrichment of marker gene sets reported in the study (ER+, HER+ and TNBC genes in breast cancer data) in gene clusters of the reduced hierarchy. The right heatmap (B) displays (1) densities of valid entries of gene clusters, (2) homogeneity *p*_*diff*_ scores of gene clusters (range in [0, 1]), (3) − *log*_10_ of hyper-geometric enrichment *P*-values of the previously reported marker genes in gene clusters (ER+, HER2+, TNBC, range in [0, 25], colors compatible with the colored dots in the left panel), (4) − *log*_10_ of FDR-adjusted hyper-geometric enrichment *P*-values of selected GO terms appeared in the left boxes in gene clusters (range in [0, 25]). The color scales of − *log*_10_ *P*-values (between 0 and 25) and density and *p*_*diff*_ (between 0 and 1) are displayed at the top of B.

We reported the following clustering analysis outcomes: (1) summaries of clustering characteristics of all datasets together (Table 2), (2) summaries of the reduced cluster hierarchies, their intra-tumoral homogeneity scores (*p*_*diff*_’s) and densities, enrichment significance with previously reported marker gene groups, and selected GO terms which are uniquely enriched in each cluster (Fig. 2 and Figs S7–S10), (3) the full gene cluster hierarchies (Fig. S11), (4) the sc-RNAseq expression profiles with genes sorted by clusters and cells sorted by tumor identities (Figs S12-–S14), (5) the full and reduced gene cluster memberships (Tables S3–S4), and the mappings from full gene clusters to reduced gene clusters (Table S5), (6) enrichment − *log*_10_(FDR-adjusted *P*-values) of GO terms in full and reduced gene clusters (Tables S6–S7), (7) uniquely enriched GO terms and their − *log*_10_(FDR-adjusted *P*-values) in full gene clusters (Table S8), (8) enrichment *P*-values of marker gene sets in full and reduced gene clusters (Tables S9A,B), (9) NSV and *p*_*diff*_ scores and densities of all full gene clusters (Table S10).

**Table 2.**
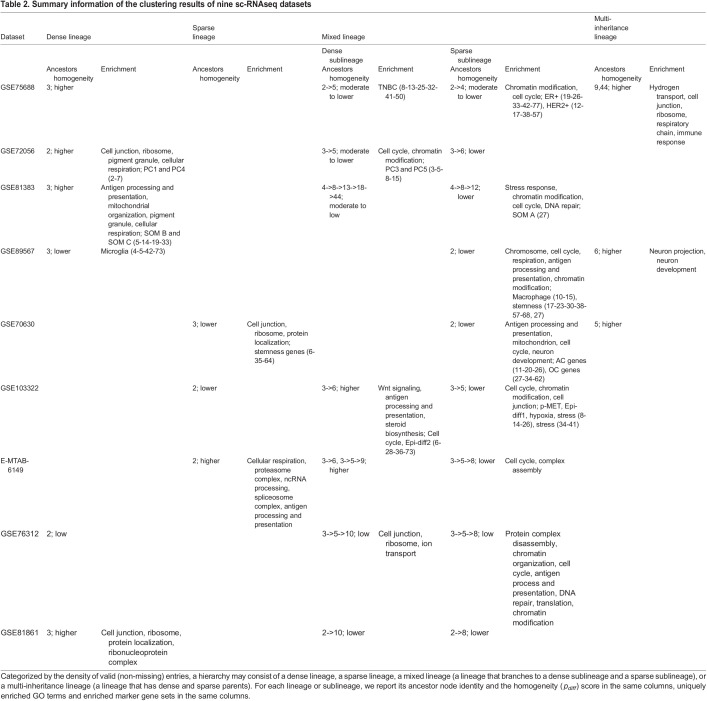
Summary information of the clustering results of nine sc-RNAseq datasets

The diverse cancer types examined in our analysis exhibit strikingly similar characteristics in terms of the functional enrichment and homogeneities of gene clusters. A major split at *k*=2 generates a relatively large lineage containing dense and sparse clusters and a relatively small lineage containing dense clusters. The dense cluster lineage typically possesses higher intra-tumoral homogeneity (higher *p*_*diff*_ scores and lower NSV scores) and enrichment with abundant functional categories such as cellular respiration, ribosome, cell junction, mitochondrion, antigen processing and presentation, and protein localization. The mixed cluster lineage often further splits into dense and sparse sublineages. The sparse cluster sublineage typically possesses lower intra-tumoral homogeneity and enrichment with scattered functional categories including cell cycle, DNA replication, and chromatin modification.

We illustrate the gene cluster characteristics with an example of the GSE75688 breast cancer data (first row of Table 2 and Fig. 2). At *k*=2 there is a large cluster (cluster 2) with mixed dense and sparse entries and a small cluster (cluster 3) with dense entries (Table S10). Cluster 2 further splits into a large sparse (4) and a small dense (5) cluster. Cluster 3 remains a lineage of small clusters. The lineages emerged from multiple clusters (starting with clusters 9 and 44, respectively) are uniquely enriched with GO terms such as hydrogen transport and mitochondrial membrane (cluster 14), cell junction and ribosomes (cluster 21) for the former (starting with cluster 9) and respiration and immune response (cluster 74) for the latter (starting with cluster 44). They also possess high levels of intra-tumoral homogeneity than other lineages. In contrast, the lineage along the sparse clusters (2-4-7 and its downstream) is uniquely enriched with chromatin modification (cluster 2) and cell cycle processes (cluster 37) and possess low levels of intra-tumoral homogeneity. The marker genes of the three breast cancer subtypes are enriched along three lineages inherited from the mixed cluster 2. ER+ genes are moderately enriched in the cluster lineage 19-26-33-42-77. HER2+ genes are moderately enriched in the cluster lineage 12-17-38-57. TNBC genes are moderately enriched in the cluster lineage 8-13-25-32-41-50. Both ER+ and HER2+ genes inherit from the sparse cluster 4, and TNBC genes inherit from the dense cluster 5.

### Pan-cancer comparison reveals consensus and specific gene clusters with differential levels of intra-tumoral homogeneity

Co-expression patterns of genes can be shared among multiple cancer types or idiosyncratic to specific cancer types. To integrate the clustering outcomes from multiple datasets, we developed an algorithm to group gene clusters into meta gene clusters that respected the hierarchical structures of individual datasets. In brief, the algorithm consists of three major parts. First, it matches paths in the hierarchies between two datasets if the clusters along the two paths considerably/moderately overlap. Paths in the hierarchy of a dataset are further merged into subgraphs if their member clusters considerably/moderately overlap and their matched paths in another dataset also considerably/moderately overlap. Second, for each dataset it constructs the membership matrix of the subgraphs over the edges in the hierarchy, applies non-negative matrix factorization multiple times to decompose those subgraphs into combinations of smaller components, and merges highly overlapped components across datasets into meta gene clusters. Third, it selects the consensus meta gene clusters over multiple trials. Detailed procedures are described in the Materials and Methods.

We generated four meta gene clusters from the gene clusters of nine datasets. Table S11 reports the meta gene cluster memberships of gene clusters. Fig. 2A and Figs S7A–S10A also display the meta gene cluster memberships by the circumference colors of the nodes in the hierarchies. Fig. S16A visualizes the overlap ratios between reduced gene clusters sorted by meta gene cluster memberships. Members of the same meta gene clusters are typically more overlapped than those between distinct meta gene clusters.

Meta gene cluster 4 is the largest and comprises primary lineages of large mixed clusters (brown nodes in Fig. 2A and Figs S7A–S10A). Meta gene cluster 3 is the second largest and comprised primary lineages of dense clusters (green nodes). Meta gene clusters 2 (red nodes) and 1 (blue nodes) are considerably smaller than meta gene clusters 3–4.

**Fig. 3.**
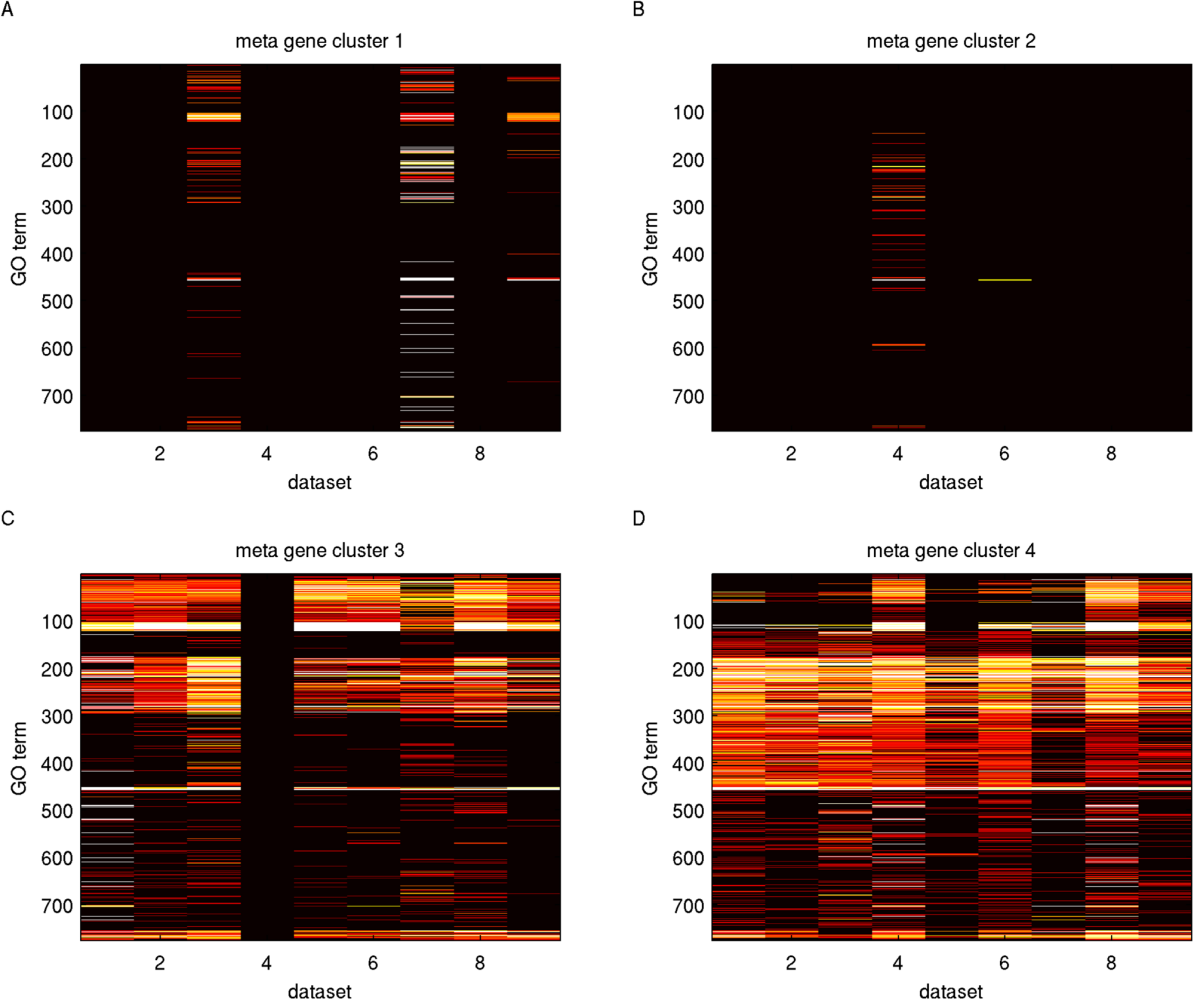
**GO term enrichment of meta gene clusters deduced from gene clusters.** Each panel displays the enrichment outcomes of one meta gene cluster over selected GO terms. The selected and sorted GO terms for meta gene clusters are identical for all panels and are reported in Table S12. In each panel, an entry visualizes the geometric mean of − *log*_10_ of FDR-adjusted *P*-values of a GO term (row) over the member clusters in a dataset (column).

We characterized meta gene clusters with enriched GO terms and intra-tumoral heterogeneity scores. We selected 775 GO terms which were enriched (FDR-adjusted *P*-values ≤ 10^−3^) in at least two datasets for at least one meta gene cluster. For each combination of meta gene cluster, dataset and GO term, we calculated the enrichment score by taking the geometric mean of enrichment *P*-values over the valid member clusters of the meta gene cluster (see the Materials and Methods). Fig. 3 and Table S12 show the enrichment scores for selected and sorted GO terms in each meta gene cluster and dataset. Sorted GO terms 1-121 are significantly enriched in meta gene cluster 3 and comprise functions pertaining to cellular respiration, antigen processing and presentation, immune responses, proton transport, and electron transport. Sorted GO terms 122-457 are significantly enriched in meta gene cluster 4 and comprise functions pertaining to cell cycle, chromosome organization, chromatin modification, DNA repair, and Golgi apparatus.

We also evaluated the intra-tumoral homogeneity scores of meta gene clusters. Since the *p*_*diff*_ scores have distinct scales among different datasets, it may not be adequate to directly average the *p*_*diff*_ scores over the member clusters. Rather, for each dataset we calculated the ranks of reduced clusters in terms of their *p*_*diff*_ scores and reported the average ranks of each meta gene cluster in Table 3. The order of meta gene clusters in terms of average intra-tumoral homogeneity ranks is 1, 3, 2 and 4. This order makes good sense since meta gene clusters 1 and 3 comprise relatively small and large dense cluster lineages, while meta gene clusters 2 and 4 comprise relatively small and large lineages of mixed clusters.

**Table 3.**
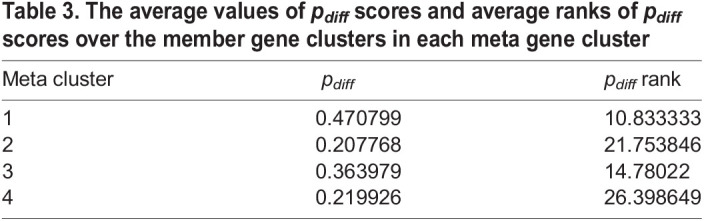
The average values of *p*_*diff*_ scores and average ranks of *p*_*diff*_ scores over the member gene clusters in each meta gene cluster

There are 97 orphan clusters that are not assigned to any meta gene clusters. For each dataset and GO term, we counted the number of orphan clusters with significant enrichment (FDR-adjusted *P*-value ≤ 10^−6^) and reported the outcomes in Table S13. Intriguingly, many enriched GO terms for those orphan clusters are related to the gene functions specific to the tissues of origins of the corresponding cancer types. Some enriched gene functions of the orphan clusters include cell junction and adhesion in GSE103322 head and neck squamous cell carcinoma data, immune responses in GSE76312 chronic myeloid leukemia data, pigmentation granule, DNA damage repair, and cell proliferation in GSE81383 melanoma data, and neuron development and projection in GSE70630 oligodendroglioma data.

### The expression patterns from bulk level and single-cell data exhibit complex relations

Although single-cell cancer transcriptomic data are increasingly prevalent, bulk level expression data are still indispensable as they are much less expensive and can cover many more patients. A critical question for combining bulk level and single-cell data is whether information derived from one type of data can be transferred to another. We addressed this question by investigating the relations of their expression patterns in both directions. First, we verified whether the combinatorial expression patterns of breast cancer subtypes in the bulk level data were preserved in sc-RNAseq data. Second, we checked whether the meta gene clusters derived from sc-RNAseq data retained coherent expressions and associations with survival times in bulk level data.

**Fig. 4.**
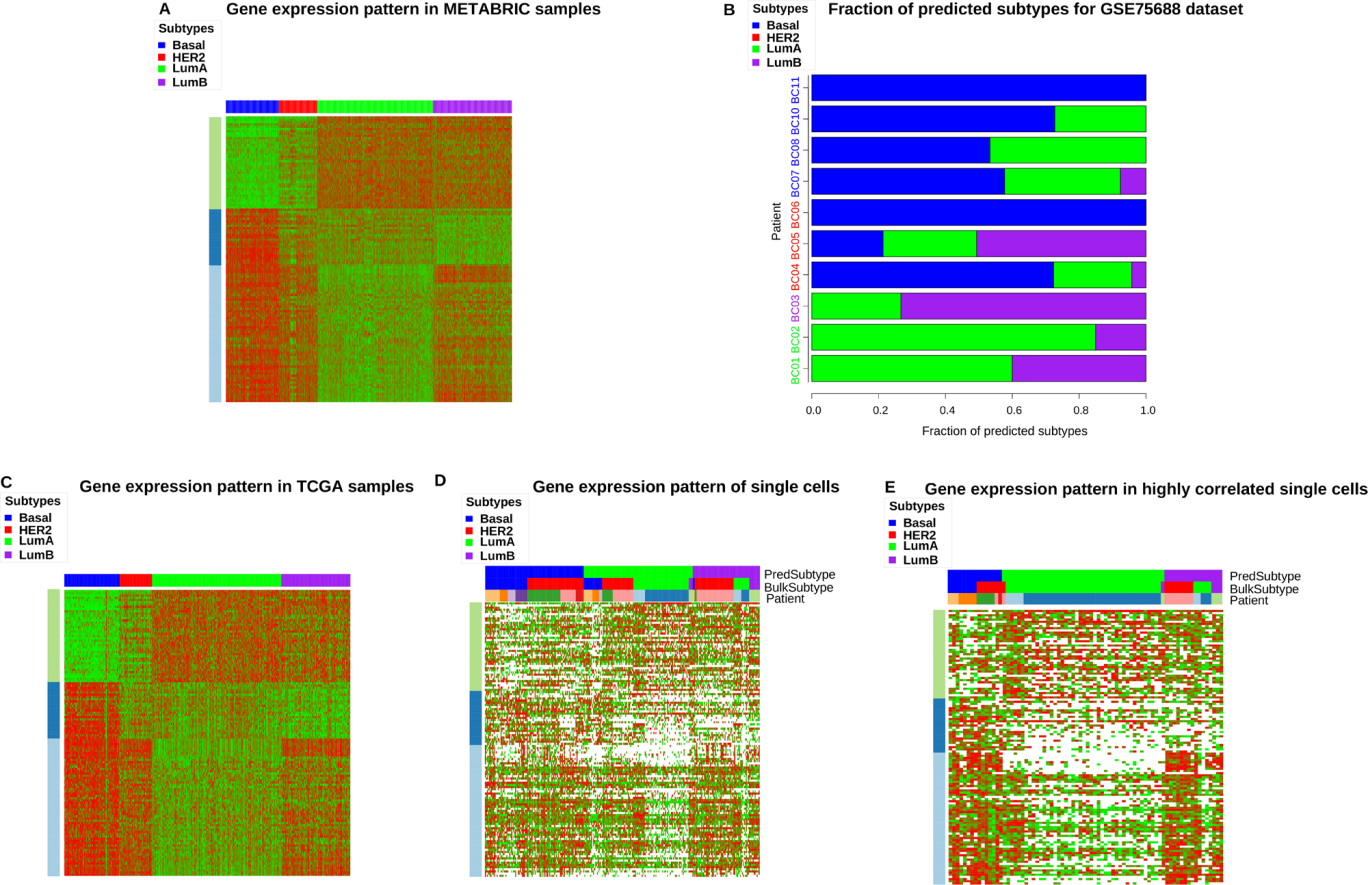
**The subtype prediction outcomes of breast cancer sc-RNAseq data.** (A) The combinatorial expression patterns of extended PAM50 genes over the four breast cancer subtypes in METABRIC data. (B) Fractions of cancer cells with predicted subtypes in each patient. The colors of the patient IDs indicate their pathological classifications. (C) The combinatorial expression patterns of extended PAM50 genes over the four breast cancer subtypes in TCGA breast cancer data. (D) The combinatorial expression patterns of extended PAM50 genes of breast cancer sc-RNAseq data. Predicted subtypes, patient (bulk) subtypes and patient IDs of single cells are annotated. (E) The combinatorial expression patterns of extended PAM50 genes of breast cancer sc-RNAseq data, including only the cells whose expression patterns are strongly correlated with the bulk level data.

Four breast cancer subtypes are demarcated by the combinatorial expression patterns of 50 genes (PAM50): Basal, Her2, Luminal A and Luminal B (Parker et al., 2009). Some PAM50 genes are no longer informative about subtype delineation in single-cell data due to their sparsity of valid entries. We extended PAM50 genes into a list of 127 genes (see the Materials and Methods) and used them to classify breast cancer subtypes in the single-cell data. The expression patterns of the extended PAM50 genes in a bulk level breast cancer transcriptomic data (METABRIC, Curtis et al., 2012) are shown in Fig. 4A. The extended PAM50 genes are divided into three groups based on their expression patterns, where groups 1–3 are enriched with estrogen response, immune response, and cell cycle process, respectively (Table S14A–C).

We developed a simple algorithm to predict the breast cancer subtypes of single-cell data (GSE75688) by comparing their extended PAM50 gene expressions with the signatures retrieved from the bulk level METABRIC data (see the Materials and Methods). Fig. 4B displays the predicted subtype compositions of cells in 10 tumors. The dominant subtypes of the tumors agree with their subtype labels according to bulk tumor assessment in 70% (seven of ten) of the tumors, which are closely aligned with the results by Chung et al. (2017).

Despite the reasonable accuracy on tumor-level prediction, the expression patterns of extended PAM50 genes in the METABRIC data are highly preserved in the bulk level TCGA BRCA data (Fig. 4C) but are not well preserved in the single-cell data (Fig. 4D). The expression vectors of about 73% of the cells are poorly correlated with the expression patterns of all subtypes (average correlation coefficient ≤ 0.1). By restricting to cells with higher average correlation coefficients (> 0.1), the single-cell expression data (Fig. 4E) more resemble the subtype expression patterns in the bulk data. There is a tradeoff between the preservation of subtype gene expression patterns and the number of cells left by varying the threshold of correlation coefficients. Fig. S15 displays the sorted sc-RNAseq expression patterns among the cells with the correlation coefficients>0.05. The expression data resembles those of all the cells (Fig. 4D), and the expression patterns of PAM50 subtypes (Fig. 4E) are no longer recognizable. We varied the correlation coefficient threshold as 0, 0.02, 0.05, 0.1, and found 100%, 92.2%, 67.7% and 27% of the cells passed the filter. No cells were left when the correlation coefficient threshold ≥ 0.2. Although the PAM50 expression patterns are poorly preserved in sc-RNAseq data with a relaxed correlation coefficient threshold, the dominant predicted subtypes in seven of ten tumors still agree with their bulk labels. In contrast, with threshold 0.1 the PAM50 expression patterns are marginally recognizable, but two tumors contain no cells and the dominant predicted subtypes in five of eight remaining tumors still agree with their bulk labels.

We further related bulk level and single-cell data by counting the overlap between PAM50 gene groups and single-cell gene clusters (Table S14D,E). There is generally poor enrichment of PAM50 gene groups in each gene cluster, implicating that the patterns of breast cancer subtype gene expressions are blurred by other processes in the single-cell data. The enrichment of breast cancer subtype gene markers of the single-cell data in gene clusters (Fig. 2) also indicates that those marker genes are enriched in clusters under the sparse lineage and possess relatively incoherent expressions.

To verify whether the observed relations between bulk and single-cell expression data in breast cancers also hold in other cancer types, we performed a similar analysis on the astrocytoma single-cell data and TCGA glioblastoma (GBM) bulk data. GBM tumors were previously categorized into four molecular subtypes (classical, mesenchymal, neural and proneural), and the marker genes of each subtype were reported (Verhaak et al., 2010). The observed correlation between the expression vectors of single cells and the expression patterns of all subtypes were also present. When the threshold of correlation coefficients varied among 0, 0.02, 0.05, 0.1 and 0.2, 100%, 89.9%, 49.9%, 10% and 0.33% of cells passed the filter. We could predict the dominant subtypes of tumors as for breast cancer data, but the prediction accuracy could not be assessed since the subtypes of astrocytoma tumors were not reported.

To address the reverse question, we downloaded and processed the mRNA expressions and survival times data of the corresponding cancer types from TCGA (Hoadley et al., 2018). The expression coherence and association with survival times of each reduced gene cluster were quantified by *p*_*diff*_ scores between the distributions of gene expression correlation coefficients/Cox regression coefficients among member genes in the cluster and among all valid genes in the data. For each TCGA cancer type, we then checked whether the members of a meta gene cluster were enriched in the top-ranking gene clusters in terms of *p*_*diff*_ scores and reported the enrichment *P*-values. Small *P*-values indicate that members in a meta gene cluster are more coherently expressed or associated with survival times relative to all reduced gene clusters in a cancer type. The detailed procedures of validations on TCGA data are described in the Materials and Methods.

Table 4 shows the enrichment *P*-values of expression coherence (4A) and survival time association (4B) for meta gene clusters. Meta gene cluster 3 possesses significant *P*-values (*P*≤0.05) in five of eight cancer types for expression coherence and positive Cox regression coefficient deviation, and almost all remaining insignificant *P*-values are also relatively small (*P*≤0.2). Other meta gene clusters either have poor *P*-values for most cancer types (such as meta gene cluster 4) or have valid entries in only a few cancer types (such as meta gene cluster 1). Meta gene cluster 3 comprise intra-tumoral homogeneous gene clusters. Strikingly, in the bulk level TCGA data these homogeneous gene clusters are coherently expressed, and their expressions are negatively associated with survival times (positive deviation of Cox regression coefficients). In contrast, in the bulk level TCGA data the heterogeneous gene clusters (meta gene cluster 4) are incoherently expressed, and their expressions are not associated with survival times.

**Table 4.**
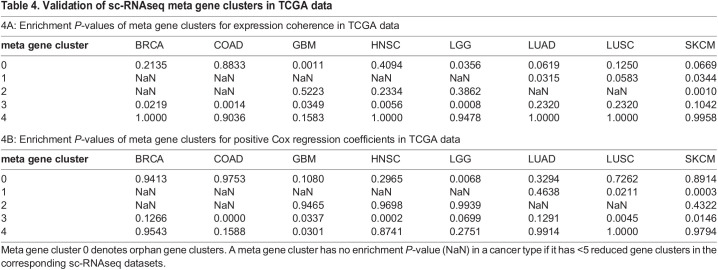
Validation of sc-RNAseq meta gene clusters in TCGA data

4B: Enrichment *P*-values of meta gene clusters for positive Cox regression coefficients in TCGA data								
**meta gene cluster**	BRCA	COAD	GBM	HNSC	LGG	LUAD	LUSC	SKCM
0	0.9413	0.9753	0.1080	0.2965	0.0068	0.3294	0.7262	0.8914
1	NaN	NaN	NaN	NaN	NaN	0.4638	0.0211	0.0003
2	NaN	NaN	0.9465	0.9698	0.9939	NaN	NaN	0.4322
3	0.1266	0.0000	0.0337	0.0002	0.0699	0.1291	0.0045	0.0146
4	0.9543	0.1588	0.0301	0.8741	0.2751	0.9914	1.0000	0.9794

## DISCUSSION

In this work, we present several bioinformatics methods for clustering analysis of cancer sc-RNAseq data. Our methods possess a number of unique features compared to prior studies. First, we explicitly quantified intra-tumoral heterogeneity/homogeneity of expression data using two measures that could be efficiently computed in high dimensional data. Second, rather than fixing the number of clusters, we clustered genes with varying numbers of clusters and represented the clustering outcomes with a compact hierarchy of clusters. Third, we aligned the cluster hierarchies from multiple datasets to form meta gene clusters and orphan gene clusters. We applied these methods to nine sc-RNAseq datasets covering seven cancer types and compared their inference results with those derived from the bulk level gene expression data. Most findings agree with previous reports about cancer sc-RNAseq data, yet some novel information also arises. The source codes of these methods are deposited in the Synapse database for public access.

Prior studies of cancer sc-RNAseq data noted higher levels of intra-tumoral heterogeneity of normal cells relative to cancer cells (Suva and Tirosh, 2019), but we also showed this trend sustained even after conditioning on normal cell types, albeit with a weaker strength (Figs S1–S6). Although the normal cell types from which cancers originate (such as luminal epithelial cells for breast cancer and colonic crypt epithelial cells for colon cancer) are not probed in the sc-RNAseq data we analyzed, in all datasets cancer cells are typically more homogeneous than almost all types of normal cells measured, suggesting that this observation is not cell type specific.

The data of colorectal cancer (GSE81861, Fig. 1F) differs from other sc-RNAseq data in our analysis as it lacks salient clustering patterns. We suspect this abnormality is partly due to the small number of cells in the data and partly due to the inferior quality of the data. All but two datasets in our study have more than 7000 valid genes and more than 500 cells (Table 1), yet the colorectal cancer data (GSE81861) has only 4683 valid genes and 480 cancer+normal cells. The colorectal cancer data is the only dataset with low numbers of valid genes and cells together. Therefore, its t-SNE projection data are likely much more sparse than other datasets.

Intra-tumoral homogeneity/heterogeneity of single cell transcriptomes may result from three interrelated processes: (1) different genetic backgrounds contribute to inter-tumoral transcriptomic differences of all cell types, (2) all cancer cells in a tumor likely descend from one malignant cell, (3) accumulated genetic/epigenetic alterations split cancer cells into subclones. We speculate that the mutational landscapes of the initiating cancer cells are likely the dominant cause of the observation that cancer cells possess more intra-tumoral homogeneity than each type of normal cells. If the genetic backgrounds of patients dominate inter-tumoral differences, then cancer cells and normal cells (of the same type) should exhibit similar levels of transcriptomic homogeneity. If tumors possess diverse subclones with distinct transcriptional signatures, then cancer cells should be more heterogeneous than each type of normal cells.

The causes of differential intra-tumoral transcriptional homogeneity among the meta gene clusters are not completely clear. A major contribution is likely expression abundance of genes. Genes with abundant/small mRNA quantities typically possess lower/higher cell-to-cell variability and lower/higher fraction of dropouts, thus constitute homogeneous/heterogeneous gene clusters such as meta gene cluster 3/meta gene cluster 4. In addition, cell cycle gene expressions (which are enriched in the meta gene cluster 4) vary with cell cycle phases in an asynchronous tumor cell population and thus may add intra-tumoral heterogeneity in sc-RNAseq data (Buettner et al., 2015; Kowalcsyk et al., 2015; Suva and Tirosh, 2019). The effect of asynchronous cell cycles on expression heterogeneity is prominent in some gene clusters which are enriched with cell cycle genes and have low *p*_*diff*_ scores but low fraction of dropouts.

Functional enrichment indicates that the coarse-level gene expression patterns of cancer cells largely inherit from the normal tissues of origin. In almost all datasets, gene clusters are enriched with four functional categories fundamental for cellular life: ribosome, respiration, RNA splicing, and cell cycle. In specific datasets, gene clusters are also enriched with functions related to their tissues of origin (such as pigmentation for melanomas, neuron projection and differentiation for brain tumors, and hematopoiesis for CML). Subtle expression patterns arising after oncogenesis – such as subclonal structures and differential epigenomic responses – may be retrieved after taking these background patterns into account.

The combinatorial expression signatures of breast cancers are highly preserved between two bulk level data (METABRIC and TCGA BRCA, figures 4A and 4C) but poorly preserved in single-cell data (Fig. 4D,E). Low concordance of the combinatorial expression patterns of marker gene groups between bulk level and single-cell level data suggests that the molecular subtypes at bulk level are aggregations/combinations of multiple refined subtypes at single-cell level or depend on interactions between tumor cells and stromal cells/immune cells microenvironment (or both). Indeed, both possible explanations were confirmed in a recent single-cell proteomic study of breast cancers (Jackson et al., 2020).

The homogeneous meta gene cluster in sc-RNAseq data retains relatively coherent expressions and negative associations with survival times in the bulk level TCGA data of the corresponding cancer types, but the heterogeneous meta gene cluster does not maintain these properties. Genes possessing intra-tumoral homogeneity in sc-RNAseq data tend to exhibit a consistent direction over the constituent cells and hence retains coherent expressions in bulk level RNAseq data. However, negative associations between the expressions of homogeneous genes and survival times in the bulk level data are less interpretable. Curiously, the homogeneous meta gene cluster is enriched with immune responses and cellular respiration, whose functions in cancers are under scrutiny in recent years.

Our study provides a crude-level characterization of intra-tumoral transcriptomic homogeneity/heterogeneity across multiple sc-RNAseq datasets. Cancer cell transcriptomes are well separated by their tumor identities, and their differences are primarily attributed to a subset of gene clusters with abundant expressions and enriched with cellular respiration, antigen processing and presentation, immune responses, cellular junction, etc. These homogeneous gene clusters retain coherent expressions and negative associations with survival times in the bulk level TCGA data. In contrast, another subset of gene clusters has low expressions in sc-RNAseq data, possesses high level intra-tumoral variability, and is enriched with cell cycle, chromosome organization, chromatin modification, and DNA repair. These heterogeneous gene clusters do not retain coherent expressions and prognostic associations in the bulk level TCGA data. This simple yet comprehensive and general picture serves as a background model that accounts for intra-tumoral homogeneity and variations of gene expressions in single-cells.

## MATERIALS AND METHODS

We describe some of the data processing and analysis methods and algorithms in this section.

### Data collection and preprocessing

Eight of the nine sc-RNAseq datasets were downloaded from the NCBI Gene Expression Omnibus (GEO) repository, and the E-MTAB-6149 NSCLC dataset was downloaded from the EBI ArrayExpress. The downloaded data were already normalized by total read counts (RPKM or FPKM). We removed the genes that contained more than 70% missing entries over all samples and passed the transformed data of the remaining genes for subsequent analysis. A bulk level breast cancer gene expression data – METABRIC – was downloaded from https://www.synapse.org/#!Synapse:syn213309. In addition, the bulk level gene expression and clinical data of eight cancer types were downloaded from the TCGA data portal https://gdc-portal.ncbi.nih.gov. They include breast invasive carcinoma (BRCA), colon adenocarcinoma (COAD), glioblastoma multiforme (GBM), head and neck squamous cell carcinoma (HNSC), brain lower grade glioma (LGG), lung adenocarcinoma (LUAD), lung squamous cell carcinoma (LUSC), and skin cutaneous melanoma (SKCM). In each dataset, the expression levels of each gene over the samples were transformed into their cumulative distribution function values. Furthermore, gene sets were downloaded from Molecular Signatures Database MSigDB (http://software.broadinstitute.org/gsea/msigdb).

### Imputing dropout entries in sc-RNAseq data

We employed a simple criterion to impute dropout entries by replacing a missing entry with the mean expression value over the valid entries of the same gene. To verify the robustness of the characteristics derived from clustering outcomes against the imputation methods, we applied two additional methods of dropout entry imputation: replacing missing entries with zeros and applying VIPER (Chen and Zhou, 2018) to the normalized data. Among the imputation methods reported in Hou et al. (2020), only VIPER fits data requirement since all other imputation programs need read counts as inputs, which are unavailable in GEO and EBI datasets. We first clustered genes on imputed data with a fixed number *k*=7 and compared the intra-tumoral homogeneity *p*_*diff*_ scores, densities of valid (non-missing) entries, and enrichment outcomes of four major functional categories (cell cycle, ribosome, respiration, and RNA splicing) in each gene cluster (Table S15). For both mean and zero imputed data, the gene clusters enriched with the four functional categories roughly follow a consistent order in terms of the *p*_*diff*_ scores or densities of valid entries: ribosome>respiration>RNA splicing>cell cycle. For VIPER-imputed data, the gene clusters enriched with ribosomes tend to have higher *p*_*diff*_ scores and densities than the gene clusters enriched with cell cycle. We then aligned gene clusters to form meta gene clusters and orphan gene clusters on each imputed data, sorted gene clusters by their meta gene cluster memberships, and counted the overlap ratios of sorted gene clusters within and between three dropout imputation methods (Fig. S16). Meta gene clusters 1, 3 and 5, 4 from zero imputation moderately overlap with meta gene clusters 3, 4 and 2 from mean imputation. There are six meta gene clusters from VIPER imputation, and meta gene clusters 2 and 5 are considerably larger than others. Meta gene cluster 2 moderately overlaps with meta gene cluster 4 from mean imputation, and meta gene cluster 5 moderately overlaps with meta gene clusters 3 and 4 from mean imputation. Meta gene cluster 2 from VIPER imputation contains primarily heterogeneous gene clusters and has a higher (less homogeneous) average rank of *p*_*diff*_ scores (22.9910), while meta gene cluster 5 from VIPER imputation contains both heterogeneous and homogeneous gene clusters and has a lower (more homogeneous) average rank of *p*_*diff*_ scores (20.7414). Meta gene cluster 6 from VIPER imputation moderately overlaps with meta gene cluster 2 from mean imputation.

### Assessing intra-tumoral heterogeneity/homogeneity

The silhouette value of a cell (Eqn 1) is determined by the average intra-tumoral and inter-tumoral distances of the expression vectors. The *p*_*diff*_ score quantifies the deviation from *p*_*intra*_ to *p*_*inter*_as *p*_*diff*_≡*P*(*X*>(*Y*+*ε*))−*P*(*X*<(*Y*−*ε*)), where random variables *X* and *Y* are sampled from *p*_*intra*_ to *p*_*inter*_, respectively. We applied the following rejection sampling procedures to draw *N* data points from a distribution *p*:

1. Start with an empty set *X*=*φ*.
2. Repeat the following steps until |*X*|=*N*.
  2.1 Uniformly draw a number *x* from the domain of *p* ([−1, 1] for correlation coefficients).
  2.2 Evaluate *p*(*x*).
  2.3 Uniformly draw a number *q*(*x*) from the interval [0, *p*_*max*_], where *p*_*max*_ is the maximum value of *p*.
  2.4 If *q*(*x*)≤*p*(*x*), then 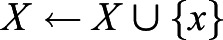.

The *p*_*diff*_ score can be directly assessed by counting the fractions of (*X*, *Y*) pairs from the sampled data satisfying the relations *X*>(*Y*+*ε*) or *X*<(*Y*−*ε*), respectively.

### Assessing intra-tumoral heterogeneity in simulated data

We generated a toy expression data comprising ten genes and 500 cells. The 500 cells were subdivided into five populations (with 100 cells per population). In each population *i*=1−5, the expression vector of each cell is ***x***=***c***_*i*_+ε, where ***c***_*i*_ is a constant vector and 
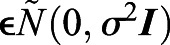
, where ***I*** is an identity matrix. Each ***c***_*i*_ contains 10 components (genes) with 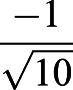 in components 2*i*−1 and 2*i* and 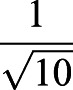 in other components, hence |***c***_*i*_|=1. The 500 cells were allocated to five tumors with three cases: (1) each tumor constituted exclusively 100 cells from one population, (2) each tumor contained 60 cells from one population and ten cells from each of the remaining populations, (3) each tumor contained 20 cells from each population. Besides NSV and *p*_*diff*_ scores, we also calculated the entropy of the expression data in each tumor. In each tumor, the marginal density of each gene was separately inferred by kernel density estimation, and the entropy of ten genes was the sum of the entropies of individual genes (assuming the genes were independent), and the average entropy over the five tumors was evaluated. We varied the standard deviation *σ* of Gaussian noise with *σ*=0.05, 0.1, 0.2, 0.3, 0.5 in simulated data and reported the three heterogeneity/homogeneity indices of the three cases in Table S1.

### Generating stable clusters using the k-means algorithm

We used the R package of ConsensusClusterPlus (Wilkerson and Hayes, 2010) to generate stable gene clusters by the k-means algorithm with a fixed *k*. In brief, it subsampled the data and ran k-means 100 times, then calculated pairwise consensus values as the fractions of runs where two genes were clustered together. An agglomerative hierarchical clustering using the distances of 1-consensus values was implemented and pruned to k groups.

### Generating the extended PAM50 gene list

The original PAM50 genes were extended into a larger gene list in order to better predict breast cancer subtypes from single-cell gene expressions. We subdivided the PAM50 genes into three groups by k-means clustering on the METABRIC gene expression data. For each gene outside the PAM50 gene list, we calculated its correlation coefficients with the PAM50 genes in both TCGA-BRCA and METABRIC data and averaged the correlation coefficients over the members of each PAM50 group. Genes were sorted by the maximum of group-level average correlation coefficients in each dataset (TCGA-BRCA and METABRIC) separately. 127 genes appeared in the top 200 genes from both datasets, and they were included in the extended PAM50 gene list.

### Predicting breast cancer subtypes from sc-RNAseq data

METABRIC samples were categorized into four subtypes: Basal, Her2, Luminal A and Luminal B. We calculated the correlation coefficients between all cells in the breast cancer sc-RNAseq data and all samples in the METABRIC RNAseq data by restricting to the 127 extended PAM50 genes. For each cell in the sc-RNAseq data, we calculated the average correlation coefficient over the METABRIC samples in each subtype and assigned it to the subtype with the maximum average correlation coefficient. We further assigned each patient in the breast cancer sc-RNAseq data to the subtype with the most abundant cells.

### Validating expression coherence and prognostic association in TCGA data

Validation on bulk level TCGA data comprises four major steps. First, we downloaded and processed the mRNA expressions and survival times data of the corresponding cancer types from TCGA. Second, for each reduced gene cluster from sc-RNAseq data we assessed its expression coherence in the corresponding TCGA data. We computed the correlation coefficients of the mRNA expressions of all pairs of genes in the data. To assess expression coherence, we compared the correlation coefficient distributions of member genes in the cluster and of all valid genes in the data and quantified the deviation between the two distributions by the *p*_*diff*_ score. Third, similar to expression coherence of each reduced gene cluster we assessed the direction and strength of association with survival times by the deviation of the Cox regression coefficient (Cox, 1972) distribution of member genes from the background distribution of all valid genes in the data (quantified again by *p*_*diff*_ scores). Fourth, for each TCGA cancer type we sorted the corresponding sc-RNAseq reduced gene clusters by their expression coherence or survival time association *p*_*diff*_ scores and calculated the *P*-values that members in a meta gene cluster were enriched in the top-ranking positions in the sorted list (Subramanian et al., 2005).

### Algorithms of simplifying and characterizing gene clusters

We describe the following algorithms below: (1) reducing a cluster hierarchy by collapsing stumps, (2) identifying the uniquely enriched clusters in a hierarchy for each GO term, (3) aligning cluster hierarchies from multiple datasets to form meta gene clusters, (4) characterizing functional enrichment and intra-tumoral heterogeneity of meta gene clusters.

### Reducing the cluster hierarchy

We propose an algorithm to reduce a cluster hierarchy by collapsing stumps in the hierarchy to single nodes. The procedures are described below.

Inputs: A directed acyclic graph *G* as the full cluster hierarchy.

Outputs: A directed acyclic graph *rG* as the reduced cluster hierarchy. The mapping *f* from nodes in *G* to nodes in *rG*.

Procedures:

1. Identify the linear nodes *L* in *G* where each node *v*∈*L* has one or no parent and one or no child.
2. Group the linear nodes in *L* to maximal chains *C*_1_, · · · , *C*_*k*_, where each chain *C*_*i*_ comprises nodes in *L* and consecutive edges in *G*, each *C*_*i*_cannot be extended by augmenting linear nodes in *L* on the top or bottom of the chain, and no two chains share common nodes.
3. Append the bottom of each chain to a node with multiple or no children (if it exists). The appended chains are called stumps.
4. Construct reduced nodes *rV* and a mapping *f* from nodes *V*∈*G* to *rV*.
  4.1 Start with *rV*=*φ*.
  4.2 For each stump *C*_*i*_, add a new node to *rV*: 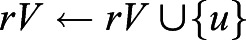. Map all nodes of *C*_*i*_ to the new node: 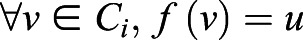.
  4.3 For each non-stump node *v*, add a new node to *rV*: 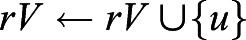, and map *v* to the new node *f*(*v*)=*u*.
5. Construct the reduced graph *rG* from *rV* and *f*. For each edge (*v*_1_, *v*_2_)∈*G*, if *u*_1_=*f*(*v*_1_)≠*f*(*v*_2_)=*u*_2_, then add edge (*u*_1_, *u*_2_) to *rG*.

We illustrate the algorithm of cluster hierarchy reduction by a toy example shown in Fig. S17. The input hierarchy comprises 11 nodes and two subgraphs. In step 1, two linear nodes *v*_4_ and *v*_8_ are identified as *L*_1_ and *L*_2_. They form the seeds of two linear chains. In step 2, the two linear chains *C*_1_ and *C*_2_ are extended by including the nonlinear nodes *v*_5_ and *v*_9_ in the bottom. In step 3, the reduced graph and the mapping are created. Nodes not belonging to maximal chains are mapped to distinct nodes in the reduced graph (e.g. *f*(*v*_1_)=*u*_1_). Nodes belonging to the same maximal chain are mapped to one node in the reduced graph (e.g. *f*(*v*_4_)=*f*(*v*_5_)=*u*_4_).

### Determining uniquely enriched GO terms

We propose an algorithm to identify the uniquely enriched clusters for each GO term. In brief, for each selected GO term, it sorts clusters by the − *log*_10_(.) transformed FDR-adjusted *P*-values and identifies the top-ranking clusters and the paths containing those clusters. It then extracts and extends the maximal path from the selected clusters and paths. The uniquely enriched cluster is the terminal node in the maximal path. The detailed procedures are described below.

Inputs: A directed graph *G* as the cluster hierarchy. The enrichment FDR-adjusted *P*-value of a GO term for each cluster.

Outputs: A cluster in *G* where the GO term is uniquely enriched, or an empty cluster *φ* if no cluster is uniquely enriched.

Procedures:

1. Transform the *P*-values by − *log*_10_(.) operation.
2. Sort clusters according to the transformed *P*-values in a descending order.
3. Enumerate all paths from the root to leaves in the hierarchy and name the list as *P*.
4. Find the list of top-ranking clusters and the paths containing those clusters.
  4.1 Generate a list *L* of clusters with top-ranking transformed *P*-values. Place the first cluster in the sorted clusters in *L*.
  4.2 Proceed with adding subsequent sorted clusters to *L* and trimming the list *P* of root-leaf paths containing all clusters in *L*, until any of the following stopping criteria are met.
    4.2.1 If the new sorted cluster *c* is not contained in any path in *P*, then stop.
    4.2.2 If *c* completes a path from the root to a leaf in *P*, then 
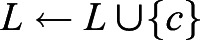
 and stop.
    4.2.3 If the transformed *P*-value of *c* is less than half of that of the last member in *L* and less than 5.0, then stop.
5. Extract the maximal path from *L* and *P*.
  5.1 If *L* comprises a complete path *p* in *P*, then report *p* as the maximal path.
  5.2 If the first sorted cluster after *L* shares the same parent as the last member of *L*, and has transformed *P*-value greater than half of that of the last member in *L* and greater than 5.0, then remove the last member of *L*.
  5.3 If all paths in *P* have holes (clusters between members of *L* and with transformed *P*-values below the minimum score in *L*), then find the maximal subpath in *P* spanned by clusters in *L*.
  5.4 Otherwise find the subpath in *P* spanned by all members in *L*.
6. Extend the maximal path if possible.
  6.1 Traverse upstream of the maximal path. If one parent of the top cluster has rank less than the max rank of the maximal path clusters+5, and score ≥5, while other parents of the top cluster do not satisfy the same criteria, then add the parent to the top of the maximal path.
  6.2 Traverse downstream of the maximal path. If one child of the bottom cluster has rank less than the max rank of the maximal path clusters+5, and score ≥5, while other children of the bottom cluster do not satisfy the same criteria, then add the child to the bottom of the maximal path.
7. Find the uniquely enriched cluster from the maximal path.
  7.1 If the maximal path exists, and the transformed score of the most downstream cluster of the maximal path ≥5, then report the most downstream cluster as the uniquely enriched cluster.
  7.2 Otherwise do not report the uniquely enriched cluster.

We illustrate the algorithm of unique enrichment determination by four toy examples shown in Fig. S18. In all examples, the top three clusters have the highest transformed *P*-values. Thus, the two root-leaf paths traversing the two branches are included in the candidate paths at steps 1–3. At step 4, the sorted cluster descends along the left branch, thus one path is removed from the candidates. Path traversing stops at step 5 for all examples. At step 5, the sorted cluster examined (red nodes) reaches a leaf (example 1, score 12), is not in the candidate path (example 2, score 12), or has considerably smaller scores than the previous node (examples 3 and 4, score 3). At step 6, the maximal path is extracted from the selected clusters and path.

### Aligning the gene cluster hierarchies across multiple datasets

We propose an algorithm to align the gene cluster hierarchies from all the datasets to form the meta gene clusters. The inputs are the reduced cluster hierarchies and the member genes of clusters for all the datasets. The outputs are the meta gene cluster labels of all clusters over all datasets. In brief, the algorithm consists of three parts. First, for each pair of datasets it matches paths in the corresponding cluster hierarchies and identifies the subgraphs spanned by the maximally matched paths. Second, it applies non-negative matrix factorization to the maximal subgraphs over multiple trials to identify the frequent subunits, and then merges highly overlapped subunits into meta gene clusters. Third, it identifies the consensus meta gene cluster over the multiple trials. Below we describe each part in detail and illustrate the procedures with toy examples and schematic diagrams.

Part 1:

Inputs: The reduced cluster hierarchies and the member genes of clusters for all the datasets.

Outputs: Subgraphs of the reduced hierarchies for all the datasets.

1. For each cluster hierarchy, enumerate all paths of clusters.
2. For each pair of datasets, identify all maximally matched path pairs.
  2.1 For each pair of clusters belonging to the two datasets (say datasets 1 and 2), determine whether they overlap according to the following criteria.
    2.1.1 If the overlap ratio of the two clusters (the Jaccard index) exceeds 0.2, then the two clusters overlap.
    2.1.2 If the overlap ratio is between 0.1 and 0.2, but cluster 2 is within the top ten dataset 2 clusters in terms of the overlap ratios with cluster 1, and cluster 1 is within the top ten dataset 1 clusters in terms of the overlap ratios with cluster 2, then the two clusters overlap.
  2.2 For each pair of paths belonging to the two datasets (e.g. 1 and 2), determine whether they are maximally matched according to the following criteria.
    2.2.1 If more than 90% of the member cluster pairs in the two paths overlap, then they are matched.
    2.2.2 If the two paths are matched but no larger paths containing those two paths are matched, then the two paths are maximally matched.
  2.3 Trim the top nodes of maximal paths if their downstream portions below the top nodes are poorly overlapped, and the matched clusters of these portions in another dataset are also poorly overlapped. By poorly overlapped we mean more than half of the cluster pairs within a collection of clusters do not overlap (2.1.1 and 2.1.2).
    2.3.1 For each target path *p*_1_ in each dataset (say dataset 1), find its matched paths in dataset 2.
    2.3.2 Find the complementary paths of *p*_1_ in dataset 1 which share the top nodes with *p*_1_, but the remaining parts do not intersect with those of *p*_1_.
    2.3.3 Find the matched paths of *p*_1_ in dataset 2.
    2.3.4 If the complementary paths (obtained in 2.3.2) and the matched paths (obtained in 2.3.3) are poorly overlapped along the clusters below the top nodes, then trim the top nodes from *p*_1_.
    2.3.5 Trim the top nodes of *p*_1_ if condition 2.3.4 is met.
  2.4 Check whether each pair of maximal paths in the same dataset are mergeable according to the following procedures.
    2.4.1 Check whether the top nodes of the two paths coincide.
    2.4.2 If so, then find their matched paths in another dataset.
    2.4.3 If the matched paths of the two paths are also considerably overlapped (more than 90% of the member cluster pairs in the two paths overlap), then mark the pair of maximal paths mergeable.
  2.5 Group the maximal paths together where all pairs of the member maximal paths are mutually mergeable. In other words, construct the cliques of maximal paths defined by the mergeable relations.
  2.6 For each dataset, construct the subgraphs spanned by the maximal path groups. Each subgraph consists of the nodes and edges contained in a maximal path group.

We illustrate the procedures of part 1 with toy examples depicted in Fig. S19. In the top row, two paths from two datasets (1→2→3→4→5 and 1^′^→2^′^→3^′^→4^′^→5^′^) are compared. Clusters 2-4 and 2′-4′ are mutually overlapped, as shown in the dotted lines in the left and the heatmap in the middle. Yet the upstream and downstream nodes of 2-4 and 2′-4′ are generally not overlapped with the counterparts in the other paths (except for the clusters 1-1′ pair). Hence the subpaths 2→3→4 and 2^′^→3^′^→4^′^ are maximally matched. In the middle row, the top node 1 appears in the paths *p*_1_ and *p*_2_ which contain disjoint downstream portions, and the matched clusters of these portions are also poorly overlapped. For instance, node 1 can be the root cluster comprising all genes, and nodes 2 and 3 are the dense and sparse clusters at k=2. Then the valid paths should exclude the top node 1. In the bottom row, two paths *p*_1_ and *p*_2_ highly overlap, and their matched paths in another dataset also highly overlap. Hence, they are merged to form a subgraph.

Part 2:

Inputs: Subgraphs of the reduced hierarchies for all the datasets.

Outputs: Meta gene clusters generated from 100 trials.

1. Repeat the following procedures 100 times for distinct random initial factorized matrices.
2. For each dataset, retrieve all maximal subgraphs and represent them as a binary matrix *A* of edge memberships. Each row represents a subgraph, and each column represents an edge in the reduced cluster hierarchy of the dataset. A unit entry denotes that an edge appears in a subgraph.
3. Vary the number of components *K* from 2 to 10. For each *K* apply Nonnegative Matrix Factorization (NMF) to find the decomposition *A*≈*W* · *H*, where entries in *W* and *H* are nonnegative.
4. Quantize *H* to a binary matrix 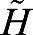. For each component (row) in *H*, keep the entries ≥ 0.1 of the maximal value and quantize them to 1, and leave the remaining entries to 0.
5. Retrieve the unique rows in 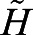 which have > 1 nonzero entry. Each unique row denotes a candidate component with at least two edges.
6. Discard the components which are disconnected, and the components that consist of multiple connected components.
7. Merge the remaining components into meta clusters.
  7.1 Establish mergeable relations for each pair of undiscarded components. If their overlap size ≥ 75% of the size of each component, then label the pair as mergeable.
  7.2 Find cliques of components according to the mergeable relations. Combine the components within each clique to form new components.

We illustrate the procedures of part 2 with toy examples depicted in Fig. S20. In the top row, 4 subgraphs *p*_1_, *p*_2_,*p*_3_,*p*_4_ span 8 edges in a cluster hierarchy. The membership matrix *A* is decomposed into *W* · *H* with two components according to NMF. *H* specifies the relaxed membership matrix of the two components. Component 1 comprises *e*_1_, *e*_2_, *e*_4_, and component 2 comprises *e*_1_, *e*_2_, *e*_3_. *W* specifies the mixture coefficients of the four subgraphs in terms of the two components. In the bottom row, the two components are merged to form a meta cluster.

Part 3:

Inputs: Meta gene clusters generated from 100 trials.

Output: The consensus meta gene clusters.

1. In each trial, build a co-occurrence matrix of gene clusters according to the meta clusters. An entry (*i*, *j*) in the co-occurrence matrix indicates whether clusters *i* and *j* appear in the same meta cluster.
2. Compute the Hamming distances between the co-occurrence matrices of each pair of trials.
3. Find the trial which has the closest total Hamming distances from all remaining trials.
4. Report the clustering outcomes of the selected trial as the meta gene clusters.
5. We manually combine a few meta gene clusters generated from the algorithm to form larger meta gene clusters.

We illustrate the procedures of part 2 with a toy example depicted in Fig. S21. In the top row, 5 NMF trials yield 5 co-occurrence matrices of meta clusters *C*_1_, *C*_2_, *C*_3_, *C*_4_, *C*_5_over ten clusters. In the middle row, the heatmap displays the Hamming distances between pairs of the co-occurrence matrices. In the bottom row, the algorithm picks the trial with the smallest overall Hamming distance with respect to all other trials (*C*_5_).

Notice that meta gene clusters are constructed by clustering the NMF components, which are collections of edges in the gene cluster hierarchies. One gene cluster may belong to multiple meta gene clusters if it belongs to multiple NMF components which are assigned to distinct meta gene clusters.

### Characterizing meta gene clusters

We characterize the meta gene clusters by both functional enrichment and intra-tumoral heterogeneity. The following procedures are adopted to calculate the enrichment FDR-adjusted *P*-values for each selected GO term in each meta gene cluster and dataset.

1. For each meta gene cluster and each dataset, identify the member reduced gene clusters.
2. Find the subgraph in the reduced cluster hierarchy spanned by the clusters obtained in the previous step. If the subgraph is disconnected, then add the minimal edges to make the subgraph connected.
3. Subtract the subgraph from the member reduced gene clusters of other meta gene clusters.
4. For each GO term, identify all paths in the subgraph satisfying the following conditions.
  4.1 The paths have ≥ 3 nodes (clusters).
  4.2 Each member cluster along the path has a FDR-adjusted *P*-value ≤ 10^−3^.
  4.3 The paths are maximal.
5. Retrieve the nodes of all the selected paths.
6. The FDR-adjusted *P*-value score of the combination of (meta gene cluster, dataset, GO term) is the geometric mean of the scores over the selected nodes.
7. Identify the GO terms whose scores ≤ 10^−3^ in at least two datasets for at least one meta gene cluster. There are 648 and 775 enriched GO terms in scenarios 1 and 2 meta gene clusters.
8. For scenario 1 clusters, meta gene clusters 1 and 5 comprise most enriched GO terms. For scenario 2 clusters, meta gene clusters 3 and 4 comprise most enriched GO terms.
9. Partition the selected GO terms into three groups for scenarios 1 and 2 clusters separately. For scenario 1 clusters, the group 1 GO terms are enriched in more datasets of meta gene cluster 1 than meta gene cluster 5. The group 2 GO terms are enriched in more datasets of meta gene cluster 5 than meta gene group 1. The remaining selected GO terms are in group 3. For scenario 2 clusters, the group 1 GO terms are enriched in more datasets of meta gene cluster 3 than meta gene cluster 4. The group 2 GO terms are enriched in more datasets of meta gene cluster 4 than meta gene group 3. The remaining selected GO terms are in group 3.
10. Sort the GO terms into the three groups for scenarios 1 and 2 clusters separately and visualize their transformed *P*-value scores in heatmaps.

To assess the average intra-tumor homogeneity of each meta gene cluster, for each dataset we sort the *p*_*diff*_ scores of the reduced clusters in a descending order and report their ranks accordingly. For a meta gene cluster, we extract the *p*_*diff*_ ranks of its member reduced clusters and report their mean.

### Validating sc-RNAseq meta gene clusters in bulk level TCGA data

We validated the sc-RNAseq meta gene clusters in the bulk level TCGA data by checking whether the member gene clusters retain coherent expressions and associations with survival times in the TCGA data. Below we depict the four steps in the validation analysis.

1. We downloaded and processed the mRNA expressions and survival times data of the corresponding cancer types from TCGA. The corresponding TCGA cancer types of sc-RNAseq datasets are reported in Table 1. All but one dataset (GSE76312 CML) have corresponding TCGA cancer types, and some datasets have multiple TCGA cancer types (such as LUAD and LUSC for E-MTAB-6149 NSCLC data).
2. For each reduced gene cluster from sc-RNAseq data, we calculated the pairwise correlation coefficients of the corresponding TCGA mRNA expression data of its member genes. We compared the distribution of these correlation coefficients (denoted by *p*_1_) with the background distribution of mRNA expression correlation coefficients among 8000 randomly selected genes (denoted by *p*_0_) from the TCGA data. The expression coherence is captured by the deviation between *p*_1_ and *p*_0_, specified by the *p*_*diff*_ score.
3. For each reduced gene cluster from sc-RNAseq data, we calculated the Cox regression coefficients of the corresponding TCGA mRNA expression data of its member genes pertaining to survival/censoring times of patients. We again compared the distribution of these Cox regression coefficients (*p*_1_) with the background distribution of the Cox regression coefficients among all genes (*p*_0_) from the TCGA data. The direction and strength of associations with survival times are captured by the *p*_*diff*_ score between *p*_1_ and *p*_0_ distributions.
4. For each TCGA cancer type we sorted the corresponding sc-RNAseq reduced gene clusters by their expression coherence or survival time association *p*_*diff*_ scores in a descending order. We then extracted the membership vector of a meta gene cluster along the sorted reduced gene clusters and generated a random walk accordingly. The random walk starts with zero, scans along the sorted clusters, and increments by one when encountering a member of the meta gene cluster. A two-dimensional curve *y*=*C*_1_(*x*) is derived from this random walk, where *x* and *y* positions of each point denote the rank of a reduced gene cluster and its random walk value. Another curve *y*=*C*_0_(*x*) is a straight line connecting the terminal points of *C*_1_(*x*). A positive deviation of *C*_1_(*x*) from *C*_0_(*x*) indicates that members of a meta gene cluster are enriched in the top-ranking clusters relative to a null model where members of a meta gene cluster are uniformly distributed along the sorted list. After normalizing both *C*_1_(*x*) and *C*_0_(*x*) by the total number of clusters, they possess characteristics of cumulative distribution functions (CDFs) where the minimum and maximum values are 0 and 1, respectively. Therefore, we quantified the deviation between *C*_1_(*x*) and *C*_0_(*x*) by the *P*-value of the one-sided Kolmogorov–Smirnov test between the two CDFs. The same procedures were employed to assess gene set enrichment *P*-values.

## Supplementary Material

Supplementary information

## References

[BIO059256C1] Akhmetzhanov, A. R., Kim, J. W., Sullivan, R., Beckman, R. A., Tamayo, P. and Yeang, C.-H. (2019). Modelling bistable tumour population dynamics to design effective treatment strategies. *J. Theor. Biol.* 474, 88-102. 10.1016/j.jtbi.2019.05.005 31077681 PMC9534689

[BIO059256C2] Beckman, R. A., Schemmann, G. S. and Yeang, C.-H. (2012). Impact of genetic dynamics and single-cell heterogeneity on development of nonstandard personalized medicine strategies for cancer. *Proc. Natl. Acad. Sci. USA* 109, 14586-14591. 10.1073/pnas.1203559109 22891318 PMC3437850

[BIO059256C3] Benjamini, Y. and Hochberg, Y. (1995). Controlling the false discovery rate: a practical and powerful approach to multiple testing. *J. R. Stat. Soc. B* 57, 289-300.

[BIO059256C4] Buettner, F., Natarajan, K. N., Casale, F. P., Proserpio, V., Scialdone, A., Theis, F. J., Teichmann, S. A., Marioni, J. C. and Stegle, O. (2015). Computational analysis of cell-to-cell heterogeneity in single-cell RNA-sequencing data reveals hidden subpopulations of cells. *Nat. Biotechnol.* 33, 155-160. 10.1038/nbt.3102 25599176

[BIO059256C5] Chao, A., C.-H. Chiu and L. Jost (2016). Phylogenetic diversity measures and their decomposition: a framework based on Hill numbers. *Biodivers. Conserv. Phylogenetic Syst.* 14, 141-172.

[BIO059256C6] Chen, M. and Zhou, X. (2018). VIPER: variability-preserving imputation for accurate gene expression recovery in single-cell RNA sequencing studies. *Genome Biol.* 19, 196. 10.1186/s13059-018-1575-1 30419955 PMC6233584

[BIO059256C7] Chen, H. J., Poran, A., Unni, A. M., Huang, S. X., Elemento, O., Snoeck, H.-W. and Varmus, H. (2019). Generation of pulmonary neuroendocrine cells and SCLC-like tumors from human embryonic stem cells. *J. Exp. Med.* 216, 674-687. 10.1084/jem.20181155 30737256 PMC6400536

[BIO059256C8] Chung, W., Eum, H. H., Lee, H.-O., Lee, K.-M., Lee, H.-B., Kim, K.-T., Ryu, H. S., Kim, S., Lee, J. E., Park, Y. H. et al. (2017). Single-cell RNA-seq enables comprehensive tumour and immune cell profiling in primary breast cancer. *Nat. Commun.* 8, 15081. 10.1038/ncomms15081 28474673 PMC5424158

[BIO059256C9] Close, H. J., Stead, L. F., Nsengimana, J., Reilly, K. A., Droop, A., Wurdak, H., Mathew, R. K., Corns, R., Newton-Bishop, J., Melcher, A. A. et al. (2020). Expression profiling of single cells and patient cohorts identifies multiple immunosuppressive pathways and an altered NK cell phenotype in glioblastoma. *Clin. Exp. Immunol.* 200, 33-44. 10.1111/cei.13403 31784984 PMC7066386

[BIO059256C10] Cox, D. R. (1972). Regression models and life-tables. *J. R. Stat. Soc. B* 32, 187-202.

[BIO059256C11] Curtis, C., Shah, S. P., Chin, S.-F., Turashvili, G., Rueda, O. M., Dunning, M. J., Speed, D., Lynch, A. G., Samarajiwa, S., Yuan, Y. et al. (2012). The genomic and transcriptomic architecture of 2000 breast tumours reveals novel subgroups. *Nature* 486, 346-352. 10.1038/nature10983 22522925 PMC3440846

[BIO059256C12] Darmanis, S., Sloan, S. A., Croote, D., Mignardi, M., Chernikova, S., Samghababi, P., Zhang, Y., Neff, N., Kowarsky, M., Caneda, C. et al. (2017). Single-cell RNA-seq analysis of infiltrating neoplastic cells at the migrating front of human glioblastoma. *Cell Rep.* 21, 1399-1410. 10.1016/j.celrep.2017.10.030 29091775 PMC5810554

[BIO059256C13] Davis, R. T., Blake, K., Ma, D., Gabra, M. B. I., Hernandez, G. A., Phung, A. T., Yang, Y., Maurer, D., Lefebvre, A. E. Y. T., Alshetaiwi, H. et al. (2020). Transcriptional diversity and bioenergetics shift in human breast cancer metastasis revealed by single-cell RNA sequencing. *Nat. Cell Biol.* 22, 310-320. 10.1038/s41556-020-0477-0 32144411

[BIO059256C14] Foerink, S. F., Sasaki, N., Lee-Six, H., Young, M. D., Alexandrov, L. B., Behjati, S., Mitchell, T. J., Grossmann, S., Lightfoot, H., Egan, D. A. et al. (2018). Intra-tumour diversification in colorectal cancer at the single-cell level. *Nature* 556, 457-462. 10.1038/s41586-018-0024-3 29643510

[BIO059256C15] Freeman, B. T., Jung, J. P. and Ogle, B. M. (2016). Single-cell RNA-seq reveals activation of unique gene groups as a consequence of stem cell-parenchymal cell fusion. *Sci. Rep.* 6, 23270. 10.1038/srep23270 26997336 PMC4800419

[BIO059256C16] Gerber, T., Willscher, E., Loeffler-Wirth, H., Hopp, L., Schadendorf, D., Schartl, M., Anderegg, U., Camp, G., Treutlein, B., Binder, H. et al. (2017). Mapping heterogeneity in patient-derived melanoma cultures by single-cell RNA-seq. *Oncotarget* 8, 846-862. 10.18632/oncotarget.13666 27903987 PMC5352202

[BIO059256C17] Gerlinger, M., Rowan, A. J., Horswell, S., Larkin, J., Endesfelder, D., Gronroos, E., Martinez, P., Matthews, N., Stewart, A., Tarpey, P. et al. (2012). Intratumor heterogeneity and branched evolution revealed by multiregion sequencing. *N Engl. J. Med.* 366, 883-892. 10.1056/NEJMoa1113205 22397650 PMC4878653

[BIO059256C18] Giustacchini, A., Thongjuea, S., Barkas, N., Woll, P. S., Povinelli, B. J., Booth, C. A. G., Sopp, P., Norfo, R., Rodriguez-Meira, A., Ashley, N. et al. (2017). Single-cell transcriptomics uncovers distinct molecular signatures of stem cells in chronic myeloid leukemia. *Nat. Med.* 23, 692-702. 10.1038/nm.4336 28504724

[BIO059256C19] Gurjao, C., Liu, D., Hofree, M., Aldubayan, S. H., Wakiro, I., Su, M.-J., Felt, K., Gjini, E., Brais, L. K., Rotem, A. et al. (2019). Intrinsic resistance to immune checkpoint blockade in a mismatch repair-deficient colorectal cancer. *Cancer Immunol. Res.* 7, 1230-1236. 10.1158/2326-6066.CIR-18-0683 31217164 PMC6679789

[BIO059256C20] Hoadley, K. A., Yau, C., Hinoue, T., Wolf, D. M., Lazar, A. J., Drill, E., Shen, R., Taylor, A. M., Cherniack, A. D., Thorsson, V. et al. (2018). Cell-of-origin patterns dominate the molecular classification of 10000 tumors from 33 types of cancer. *Cell* 173, 291-304. 10.1016/j.cell.2018.03.022 29625048 PMC5957518

[BIO059256C21] Horning, A. M., Wang, Y., Lin, C.-K., Louie, A. D., Jadhav, R. R., Hung, C.-N., Wang, C.-M., Lin, C.-L., Kirma, N. B., Liss, M. A. et al. (2018). Single-cell RNA-seq reveals a subpopulation of prostate cancer cells with enhanced cell-cycle-related transcription and attenuated androgen response. *Cancer Res.* 78, 853-864. 10.1158/0008-5472.CAN-17-1924 29233929 PMC5983359

[BIO059256C22] Hou, W., Ji, Z., Ji, H. and Hicks, S. C. (2020). A systematic evaluation of single-cell RNA-sequencing imputation methods. *Genome Biol.* 21, 218. 10.1186/s13059-020-02132-x 32854757 PMC7450705

[BIO059256C23] Jackson, H. W., Fischer, J. R., Zanotelli, V. R. T., Ali, H. R., Mechera, R., Soysal, S. D., Moch, H., Muenst, S., Varga, Z., Weber, W. P. et al. (2020). The single-cell pathology landscape of breast cancer. *Nature* 578, 615-620. 10.1038/s41586-019-1876-x 31959985

[BIO059256C24] Jang, B. S., Han, W. and Kim, I. A. (2020). Tumor mutation burden, immune checkpoint crosstalk and radiosensitivity in single-cell RNA sequencing data of breast cancer. *Radiother. Oncol.* 142, 202-209. 10.1016/j.radonc.2019.11.003 31767471

[BIO059256C25] Kashima, Y., Suzuki, A., Liu, Y., Hosokawa, M., Matsunaga, H., Shirai, M., Arikawa, K., Sugano, S., Kohno, T., Takeyama, H. et al. (2018). Combinatory use of distinct single-cell RNA-seq analytical platforms reveals the heterogeneous transcriptome response. *Sci. Rep.* 8, 3482. 10.1038/s41598-018-21161-y 29472726 PMC5823859

[BIO059256C26] Kester, L. and Van Oudenaarden, A. (2018). Single-cell transcriptomics meets lineage tracing. *Cell Stem Cell* 23, 166-179. 10.1016/j.stem.2018.04.014 29754780

[BIO059256C27] Kim, K. T., Lee, H. W., Lee, H.-O., Kim, S. C., Seo, Y. J., Chung, W., Eum, H. H., Nam, D.-H., Kim, J., Joo, K. M. et al. (2015). Single-cell mRNA sequencing identifies subclonal heterogeneity in anti-cancer drug responses of lung adenocarcinoma cells. *Genome Biol.* 16, 127. 10.1186/s13059-015-0692-3 26084335 PMC4506401

[BIO059256C28] Kim, K., Park, S., Park, S. Y., Kim, G., Park, S. M., Cho, J.-W., Kim, D. H., Park, Y. M., Koh, Y. W., Kim, H. R. et al. (2020). Single-cell transcriptome analysis reveals TOX as a promoting factor for T cell exhaustion and a predictor for anti-PD-1 response in human cancer. *Genome Med.* 12, 22. 10.1186/s13073-020-00722-9 32111241 PMC7048139

[BIO059256C29] Kowalcsyk, M. S., Tirosh, I., Heckl, D., Rao, T. N., Dixit, A., Haas, B. J., Schneider, R. K., Wagers, A. J., Ebert, B. L. and Regev, A. (2015). Single-cell RNA-seq reveals changes in cell cycle and differentiation programs upon aging of hematopoietic stem cells. *Genome Res.* 25, 1860-1872. 10.1101/gr.192237.115 26430063 PMC4665007

[BIO059256C30] Lambrechts, D., Wauters, E., Boeckx, B., Aibar, S., Nittner, D., Burton, O., Bassez, A., Decaluwé, H., Pircher, A., Van Den Eynde, K. et al. (2018). Phenotype molding of stromal cells in the lung tumor environment. *Nat. Med.* 24, 1277-1289. 10.1038/s41591-018-0096-5 29988129

[BIO059256C31] Lei, B., Zhang, X.-y, Zhou, J.-p, Mu, G.-n, Li, Y.-w, Zhang, Y.-x and Pang, D. (2016). Transcriptome sequencing of HER2-positive breast cancer stem cells identifies potential prognostic marker. *Tumor Biol.* 37, 14757-14764. 10.1007/s13277-016-5351-0 27629143

[BIO059256C32] Li, H., Courtois, E. T., Sengupta, D., Tan, Y., Chen, K. H., Goh, J. J. L., Kong, S. L., Chua, C., Hon, L. K., Tan, W. S. et al. (2017). Reference component analysis of single-cell transcriptomes elucidates cellular heterogeneity in human colorectal tumors. *Nat. Genet.* 49, 708-718. 10.1038/ng.3818 28319088

[BIO059256C33] Li, S. C., Stucky, A., Chen, X., Kabeer, M. H., Loudon, W. G., Plant, A. S., Torno, L., Nangia, C. S., Cai, J., Zhang, G. et al. (2018). Single-cell transcriptomes reveal the mechanism for a breast cancer prognostic gene panel. *Oncotarget* 9, 33290-33301. 10.18632/oncotarget.26044 30279960 PMC6161791

[BIO059256C34] Lu, Y. C., Jia, L., Zheng, Z., Tran, E., Robbins, P. F. and Rosenberg, S. A. (2019). Single-cell transcriptome analysis reveals gene signatures associated with T-cell persistence following adoptive cell therapy. *Cancer Immunol. Res.* 7, 1824-1836. 10.1158/2326-6066.CIR-19-0299 31484655 PMC6825592

[BIO059256C35] Nguyen, A., Yoshida, M., Goodarzi, H. and Tavazoie, S. F. (2016). Highly variable cancer subpopulations that exhibit enhanced transcriptome variability and metastatic fitness. *Nat. Commun.* 7, 11246. 10.1038/ncomms11246 27138336 PMC4857405

[BIO059256C36] Park, Y., Lim, S., Nam, J.-W. and Kim, S. (2016). Measuring intratumor heterogeneity by network entropy using RNA-seq data. *Sci. Rep.* 6, 37767. 10.1038/srep37767 27883053 PMC5121893

[BIO059256C37] Parker, J. S., Mullins, M., Cheang, M. C. U., Leung, S., Voduc, D., Vickery, T., Davies, S., Fauron, C., He, X., Hu, Z. et al. (2009). Supervised risk predictor of breast cancer based on intrinsic subtypes. *J. Clin. Oncol.* 27, 1160-1167. 10.1200/JCO.2008.18.1370 19204204 PMC2667820

[BIO059256C38] Peired, A. J., Antonelli, G., Angelotti, M. L., Allinovi, M., Guzzi, F., Sisti, A., Semeraro, R., Conte, C., Mazzinghi, B., Nardi, S. et al. (2020). Acute kidney injury promotes development of papillary renal cell adenoma and carcinoma from renal progenitor cells. *Sci. Transl. Med.* 12, eaaw6003. 10.1126/scitranslmed.aaw6003 32213630

[BIO059256C39] Peixoto, P., Etcheverry, A., Aubry, M., Missey, A., Lachat, C., Perrard, J., Hendrick, E., Delage-Mourroux, R., Mosser, J., Borg, C. et al. (2019). EMT is associated with an epigenetic signature of ECM remodeling genes. *Cell Death Dis.* 10, 205. 10.1038/s41419-019-1397-4 30814494 PMC6393505

[BIO059256C40] Peng, J., Sun, B.-F., Chen, C.-Y., Zhou, J.-Y., Chen, Y.-S., Chen, H., Liu, L., Huang, D., Jiang, J., Cui, G.-S. et al. (2019). Single-cell RNA-seq highlights intra-tumoral heterogeneity and malignant progression in pan-cancer ductal adenocarcinoma. *Cell Res.* 29, 725-738. 10.1038/s41422-019-0195-y 31273297 PMC6796938

[BIO059256C41] Praktiknjo, S. D., Obermayer, B., Zhu, Q., Fang, L., Liu, H., Quinn, H., Stoeckius, M., Kocks, C., Birchmeier, W. and Rajewsky, N. (2020). Tracing tumorigenesis in a solid tumor model at single-cell resolution. *Nat. Commun.* 11, 991. 10.1038/s41467-020-14777-0 32080185 PMC7033116

[BIO059256C42] Puram, S. V., Tirosh, I., Parikh, A. S., Patel, A. P., Yizhak, K., Gillespie, S., Rodman, C., Luo, C. L., Mroz, E. A., Emerick, K. S. et al. (2017). Single-cell transcriptomic analysis of primary and metastatic tumor ecosystems in head and neck cancer. *Cell* 171, 1611-1624. 10.1016/j.cell.2017.10.044 29198524 PMC5878932

[BIO059256C43] Rousseeuw, P. J. (1987). Silhouettes: a graphical aid to the interpretation and validation of cluster analysis. *Comput. Appl. Math.* 20, 53-65. 10.1016/0377-0427(87)90125-7

[BIO059256C44] Simpson, E. H. (1949). Measurement of diversity. *Nature* 163, 688. 10.1038/163688a0

[BIO059256C45] Subramanian, A., Tamayo, P., Mootha, V. K., Mukherjee, S., Ebert, B. L., Gillette, M. A., Paulovich, A., Pomeroy, S. L., Golub, T. R., Lander, E. S. et al. (2005). Gene set enrichment analysis: a knowledge-based approach for interpreting genome-wide expression profiles. *Proc. Natl. Acad. Sci. U.S.A.* 102, 15545-15550. 10.1073/pnas.0506580102 16199517 PMC1239896

[BIO059256C46] Suva, M. L. and Tirosh, I. (2019). Single-cell RNA sequencing in cancer: lessons learned and emerging challenges. *Mol. Cell* 75, 7-12. 10.1016/j.molcel.2019.05.003 31299208

[BIO059256C47] Suzuki, A., Matsushima, K., Makinoshima, H., Sugano, S., Kohno, T., Tsuchihara, K. and Suzuki, Y. (2015). Single-cell analysis of lung adenocarcinoma cell lines reveals diverse expression patterns of individual cells invoked by a molecular target drug treatment. *Genome Biol.* 16, 66. 10.1186/s13059-015-0636-y 25887790 PMC4450998

[BIO059256C48] Teschendorff, A. E. and Enver, T. (2017). Single-cell entropy for accurate estimation of differentiation potency from a cell's transcriptome. *Nat. Commun.* 8, 15599. 10.1038/ncomms15599 28569836 PMC5461595

[BIO059256C49] Tirosh, I., Venteicher, A. S., Hebert, C., Escalante, L. E., Patel, A. P., Yizhak, K., Fisher, J. M., Rodman, C., Mount, C., Filbin, M. G. et al. (2016a). Single-cell RNA-seq supports a developmental hierarchy in human oligodendroglioma. *Nature* 539, 309-313. 10.1038/nature20123 27806376 PMC5465819

[BIO059256C50] Tirosh, I., Izar, B., Prakadan, S. M., Wadsworth, M. H., Treacy, D., Trombetta, J. J., Rotem, A., Rodman, C., Lian, C., Murphy, G. et al. (2016b). Dissecting the multicellular ecosystem of metastatic melanoma by single-cell RNA-seq. *Science* 352, 189-196. 10.1126/science.aad0501 27124452 PMC4944528

[BIO059256C51] Tsoucas, D. and Yuan, G. C. (2017). Recent progress in single-cell cancer genomics. *Curr. Opin. Genet. Dev.* 42, 22-32. 10.1016/j.gde.2017.01.002 28126650 PMC5446798

[BIO059256C52] Van Der Maaten, L. J. P. and Hinton, G. E. (2008). Visualizing high-dimensional data using t-SNE. *J. Mach. Learn. Res.* 9, 2579-2605.

[BIO059256C54] Venteicher, A. S., Tirosh, I., Hebert, C., Yizhak, K., Neftel, C., Filbin, M. G., Hovestadt, V., Escalante, L. E., Shaw, M. K. L., Rodman, C. et al. (2017). Decoupling genetics, lineages, and microenvironment in IDH-mutant gliomas by single-cell RNA-seq. *Science* 355, eaai8478. 10.1126/science.aai8478 28360267 PMC5519096

[BIO059256C55] Verhaak, R. G., Hoadley, K. A., Purdom, E., Wang, V., Qi, Y., Wilkerson, M. D., Miller, C. R., Ding, L., Golub, T., Mesirov, J. P. et al. (2010). Integrated genomic analysis identifies clinical relevant subtypes of glioblastoma characterized by abnormalities in PDGFRA, IDH1, and NF1. *Cancer Cell* 17, 98-110. 10.1016/j.ccr.2009.12.020 20129251 PMC2818769

[BIO059256C56] Vinogradov, A. E. and Anatskaya, O. (2020). Cell-cycle dependence of transcriptome gene modules: comparison of regression lines. *FEBS J.* 287, 4427-4439. 10.1111/febs.15257 32083797

[BIO059256C57] Wang, Y., Waters, J., Leung, M. L., Unruh, A., Roh, W., Shi, X., Chen, K., Scheet, P., Vattathil, S., Liang, H. et al. (2014). Clonal evolution in breast cancer revealed by single nucleus genome sequencing. *Nature* 512, 155-160. 10.1038/nature13600 25079324 PMC4158312

[BIO059256C58] Wilkerson, M. D. and Hayes, D. N. (2010). ConsensClusterPlus: a class discovery tool with confidence assessments and item tracking. *Bioinformatics* 26, 1572-1573. 10.1093/bioinformatics/btq170 20427518 PMC2881355

[BIO059256C59] Wu, H., Yu, J., Li, Y., Hou, Q., Zhou, R., Zhang, N., Jing, Z., Jiang, M., Li, Z., Hua, Y. et al. (2018). Single-cell RNA sequencing reveals diverse intratumoral heterogeneities and gene signatures of two types of esophageal cancers. *Cancer Letter* 438, 133-143. 10.1016/j.canlet.2018.09.017 30223068

[BIO059256C60] Wu, H., Li, Y., Hou, Q., Zhou, R., Li, Z., Wu, S., Yu, J. and Jiang, M. (2019). Single-cell intratumoral stemness analysis reveals the involvement of cell cycle and DNA damage repair in two different types of esophageal cancer. *Oncol. Rep.* 41, 3201-3208. 10.3892/or.2019.7117 31002369 PMC6489016

[BIO059256C61] Young, M. D., Mitchell, T. J., Vieira Braga, F. A., Tran, M. G. B., Stewart, B. J., Ferdinand, J. R., Collord, G., Botting, R. A., Popescu, D.-M., Loudon, K. W. et al. (2018). Single-cell transcriptomes from human kidneys reveal the cellular identity of renal tumors. *Science* 361, 594-599. 10.1126/science.aat1699 30093597 PMC6104812

[BIO059256C62] Yue, M., Kim, J. C., Wilson, G. W., Ng, K., Figueroa, E. F., O′Kane, G. M., Connor, A. A., Denroche, R. E., Grant, R. C., Mcleod, J. et al. (2020). Transcription phenotypes of pancreatic cancer are driven by genomic events during tumor evolution. *Nat. Genet.* 52, 231-240. 10.1038/s41588-019-0566-9 31932696

[BIO059256C63] Zhang, X., Zhang, M., Hou, Y., Xu, L., Li, W., Zou, Z., Liu, C., Xu, A. and Wu, S. (2016). Single-cell analyses of transcriptional heterogeneity in squamous cell carcinoma of urinary bladder. *Oncotarget* 7, 66069-66076. 10.18632/oncotarget.11803 27602771 PMC5323215

[BIO059256C64] Zhang, W., Bouchard, G., Yu, A., Shafiq, M., Jamali, M., Shrager, J. B., Ayers, K., Bakr, S., Gentles, A. J., Diehn, M. et al. (2018). GFPT2-expressing cancer-associated fibroblasts mediate metabolic reprogramming in human lung adenocarcinoma. *Cancer Res.* 78, 3445-3457. 10.1158/0008-5472.CAN-17-2928 29760045 PMC6030462

[BIO059256C65] Zhang, P., Yang, M., Zhang, Y., Xiao, S., Lai, X., Tan, A., Du, S. and Li, S. (2019). Dissecting the single-cell transcriptome network underlying gastric premalignant lesions and early gastric cancer. *Cell Rep.* 27, 1934-1947. 10.1016/j.celrep.2019.04.052 31067475

[BIO059256C66] Zhang, Y., Song, J., Zhao, Z., Yang, M., Chen, M., Liu, C., Ji, J. and Zhu, D. (2020). Single-cell transcriptome analysis reveals tumor immune microenvironment heterogeneity and granulocytes enrichment in colorectal cancer liver metastasis. *Cancer Letter* 470, 84-94. 10.1016/j.canlet.2019.10.016 31610266

[BIO059256C67] Zhao, Q., Eichten, A., Parveen, A., Adler, C., Huang, Y., Wang, W., Ding, Y., Adler, A., Nevins, T., Ni, M. et al. (2018). Single-cell transcriptome analyses reveal endothelial cell heterogeneity in tumors and changes following antiangiogenic treatment. *Cancer Res.* 78, 2370-2382. 10.1158/0008-5472.CAN-17-2728 29449267

[BIO059256C68] Zheng, H., Pomyen, Y., Hernandez, M. O., Li, C., Livak, F., Tang, W., Dang, H., Greten, T. F., Davis, J. L., Zhao, Y. et al. (2018). Single cell analysis reveals cancer stem cell heterogeneity in hepatocellular carcinoma. *Hepatology* 68, 127-140. 10.1002/hep.29778 29315726 PMC6033650

[BIO059256C69] Zhu, D., Zhao, Z., Cui, G., Chang, S., Hu, L., See, Y. X., Lim, M. G. L., Guo, D., Chen, X., Poudel, B. et al. (2018). Single-cell transcriptome analysis reveals estrogen signaling coordinately augments one-carbon, polyamine, and purine in breast cancer. *Cell Rep.* 25, 2285-2298.e4. 10.1016/j.celrep.2018.10.093 30463022

