# Supplementary material for: Characterization of gene cluster heterogeneity in single-cell transcriptomic data within and across cancer types
Source: Biol Open. 2022 Jun 23;11(6):bio059256. doi: 10.1242/bio.059256 (PMC9235070; doi:10.1242/bio.059256)
Supplement: Supplementary information [file biolopen-11-059256-s1.pdf]

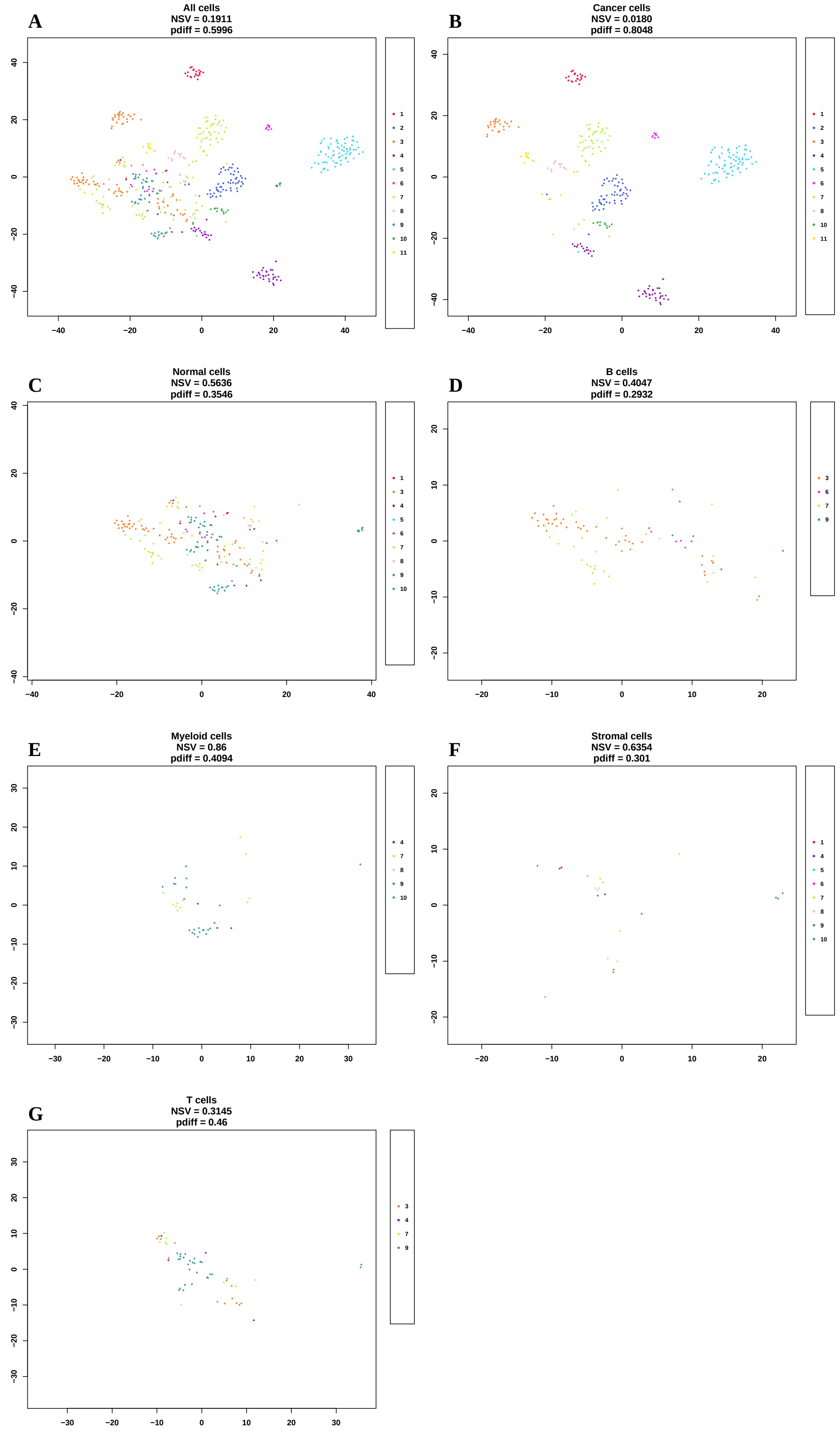
A
All cells
NSV = 0.1911
pdiff = 0.5996
B
Cancer cells
NSV = 0.0180
pdiff = 0.8048
C
Normal cells
NSV = 0.5636
pdiff = 0.3546
D
B cells
NSV = 0.4047
pdiff = 0.2932
E
Myeloid cells
NSV = 0.86
pdiff = 0.4094
F
Stromal cells
NSV = 0.6354
pdiff = 0.301
G
T cells
NSV = 0.3145
pdiff = 0.46

Fig. S1. t-SNE plots SE75688 breast cancer sc-RNAseq data. Panels display all cells together, cancer and normal cells alone, and each type of normal cells separately. Average NSV fractions and p_{diff} scores are reported.

Biology Open • Supplementary information

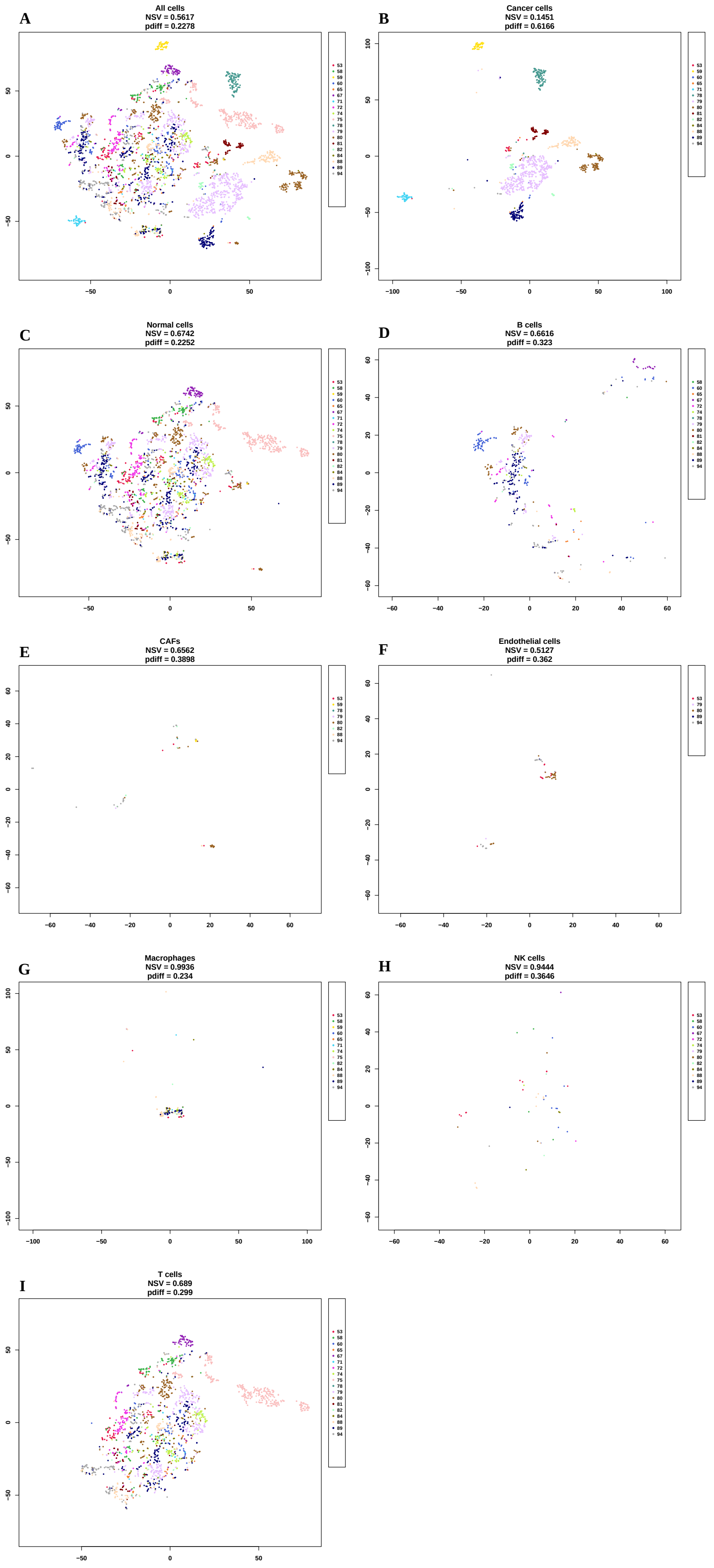

Fig. S2. t-SNE plots of GSE72056 melanoma sc-RNAseq data. Legend follows Supplementary Figure S1.

Biology Open • Supplementary information

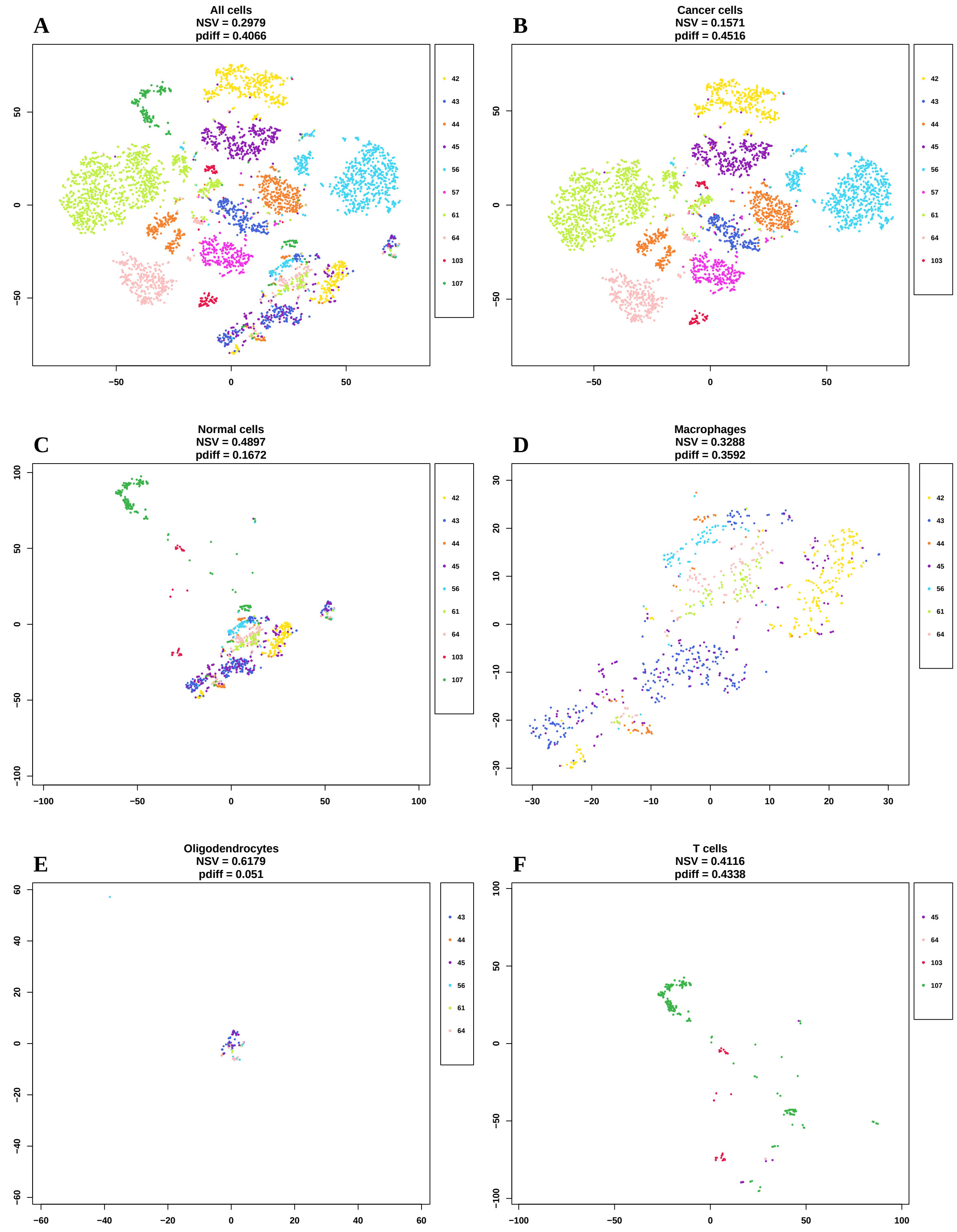

Fig. S3. t-SNE plots of GSE89567 astrocytoma sc-RNAseq data. Legend follows Supplementary Figure S1.

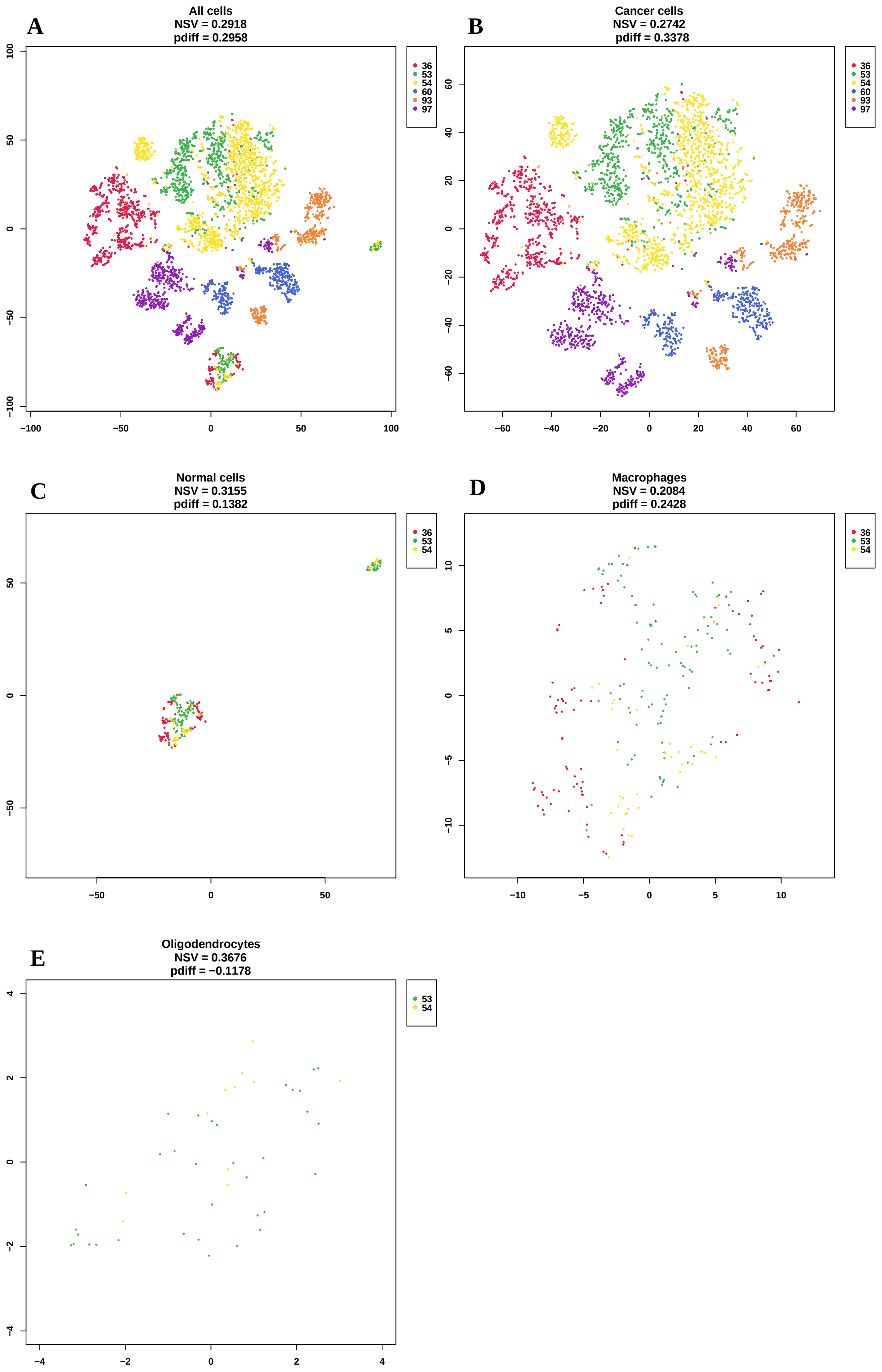

Fig. S4. t-SNE plots SE70630 oligodendroglioma sc-RNAseq data. Legend follows Supplementary Figure S1.

Biology Open • Supplementary information

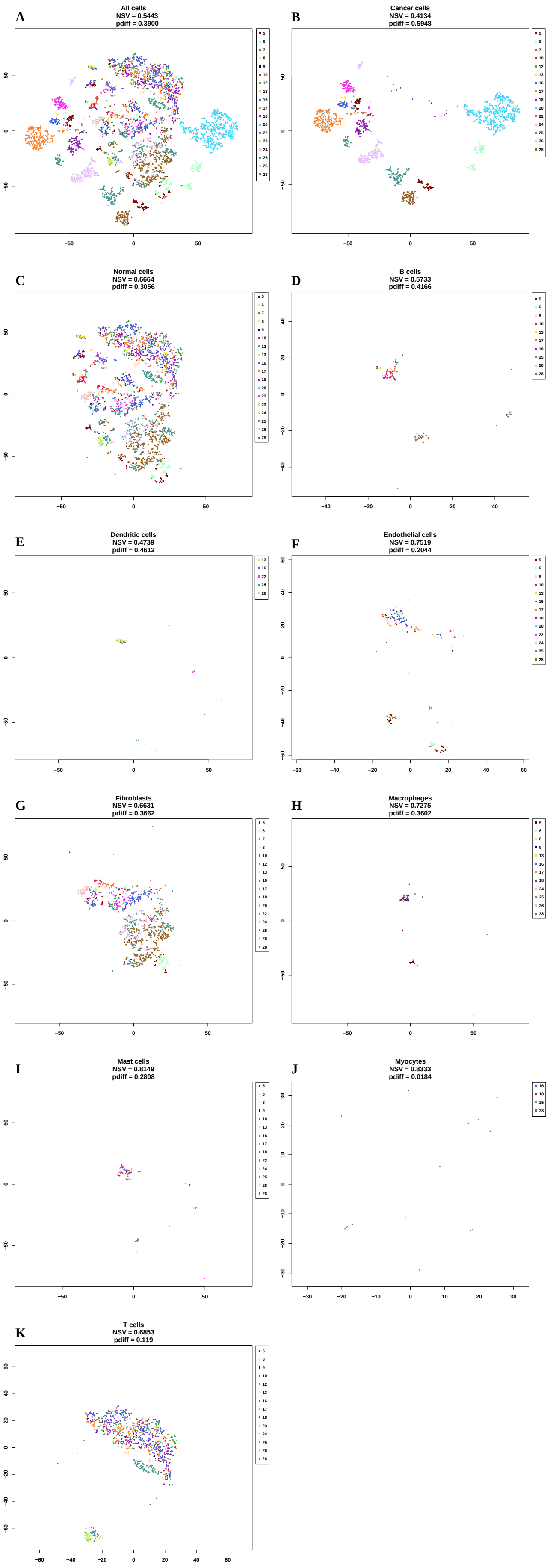

Fig. S5. t-SNE plots of GSE103322 head and neck squamous cell carcinoma sc-RNAseq data. Legend follows Supplementary Figure S1.

Biology Open • Supplementary information

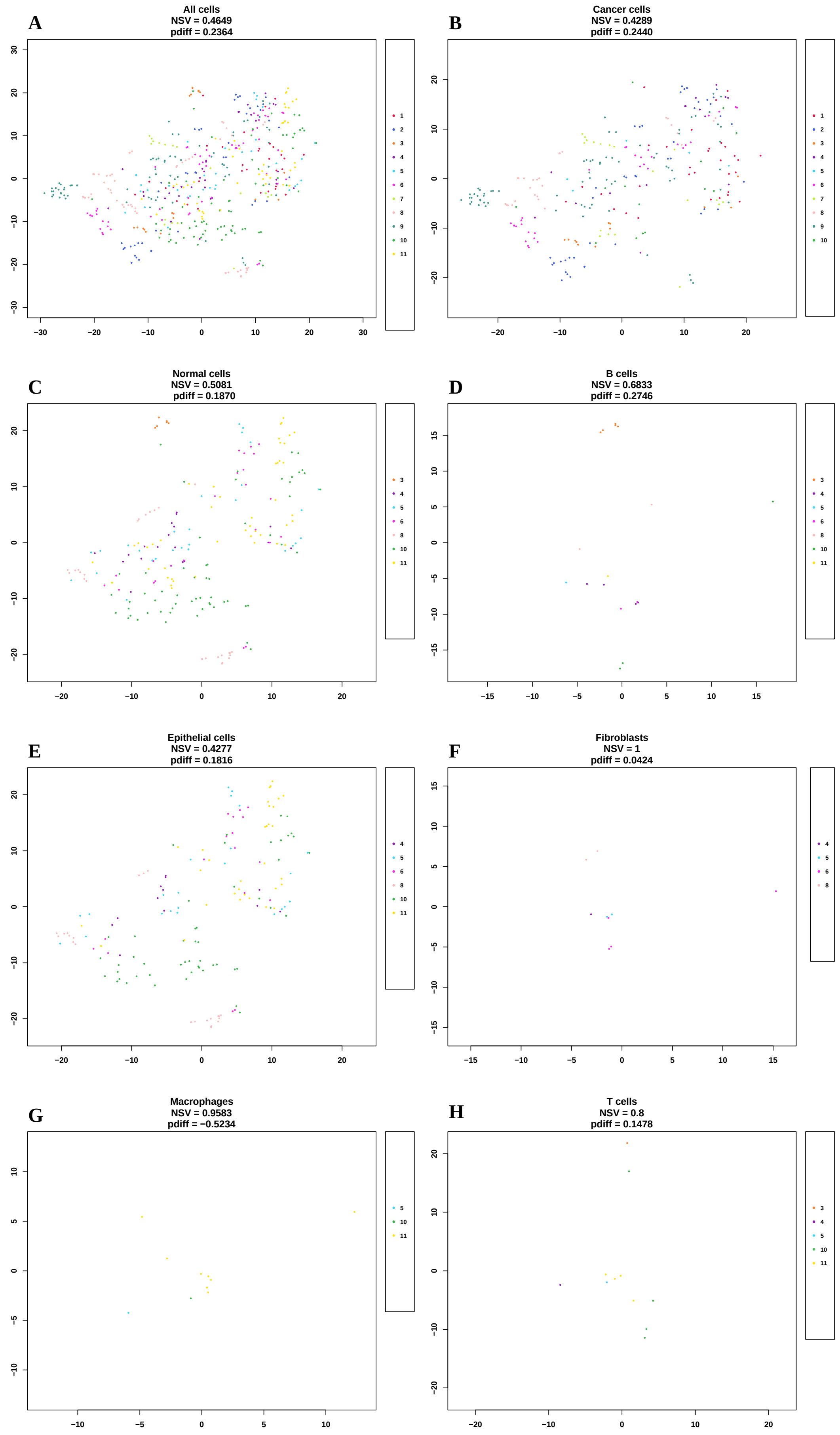

Fig. S6. t-SNE plots of GSE81861 colorectal cancer sc-RNAseq data. Legend follows Supplementary Figure S1.

Biology Open • Supplementary information

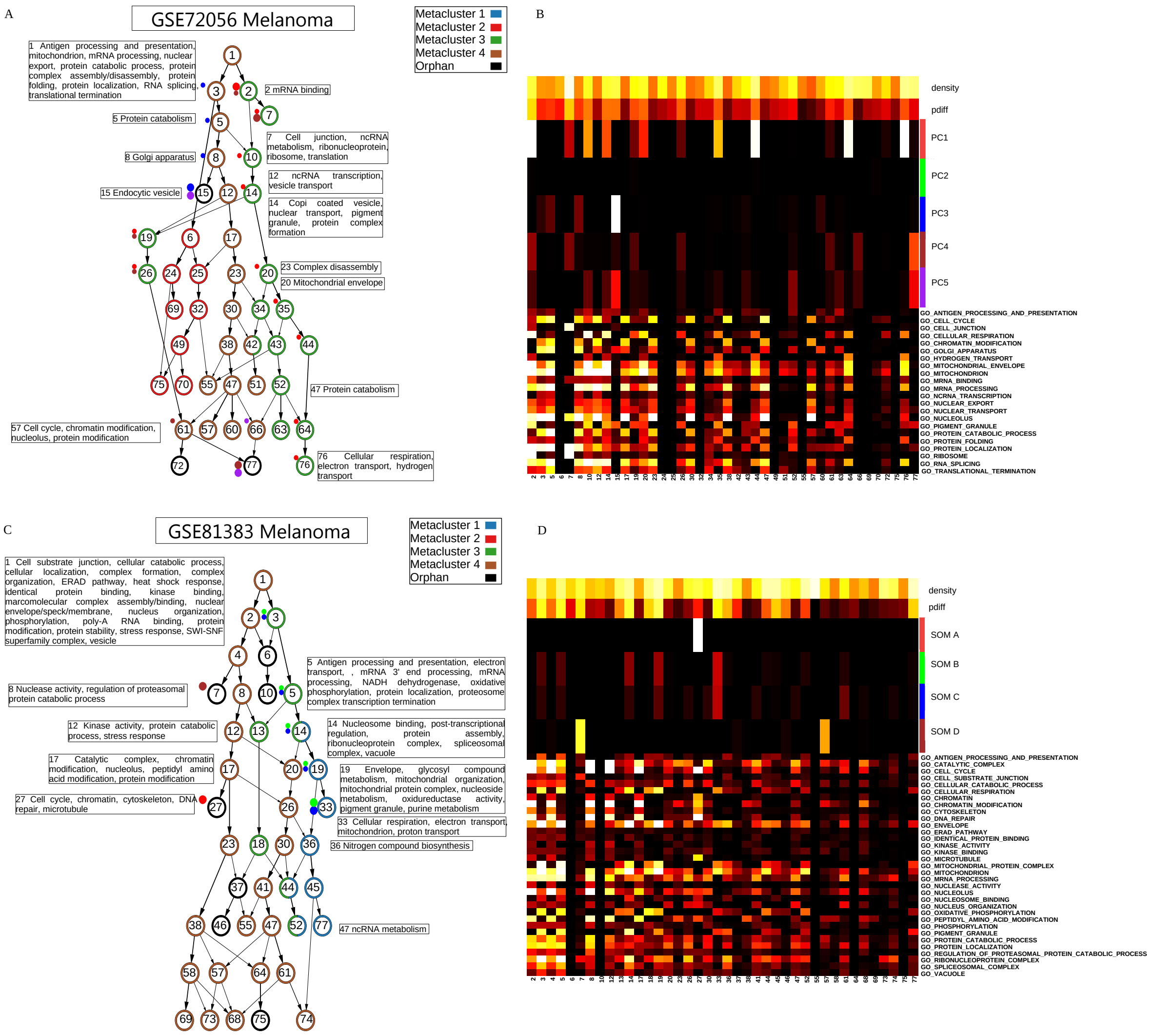

Fig. S7. Summary information of clustering results of GSE72056 and GSE81383 melanoma data. Legend follows Figure 2.

Biology Open • Supplementary information

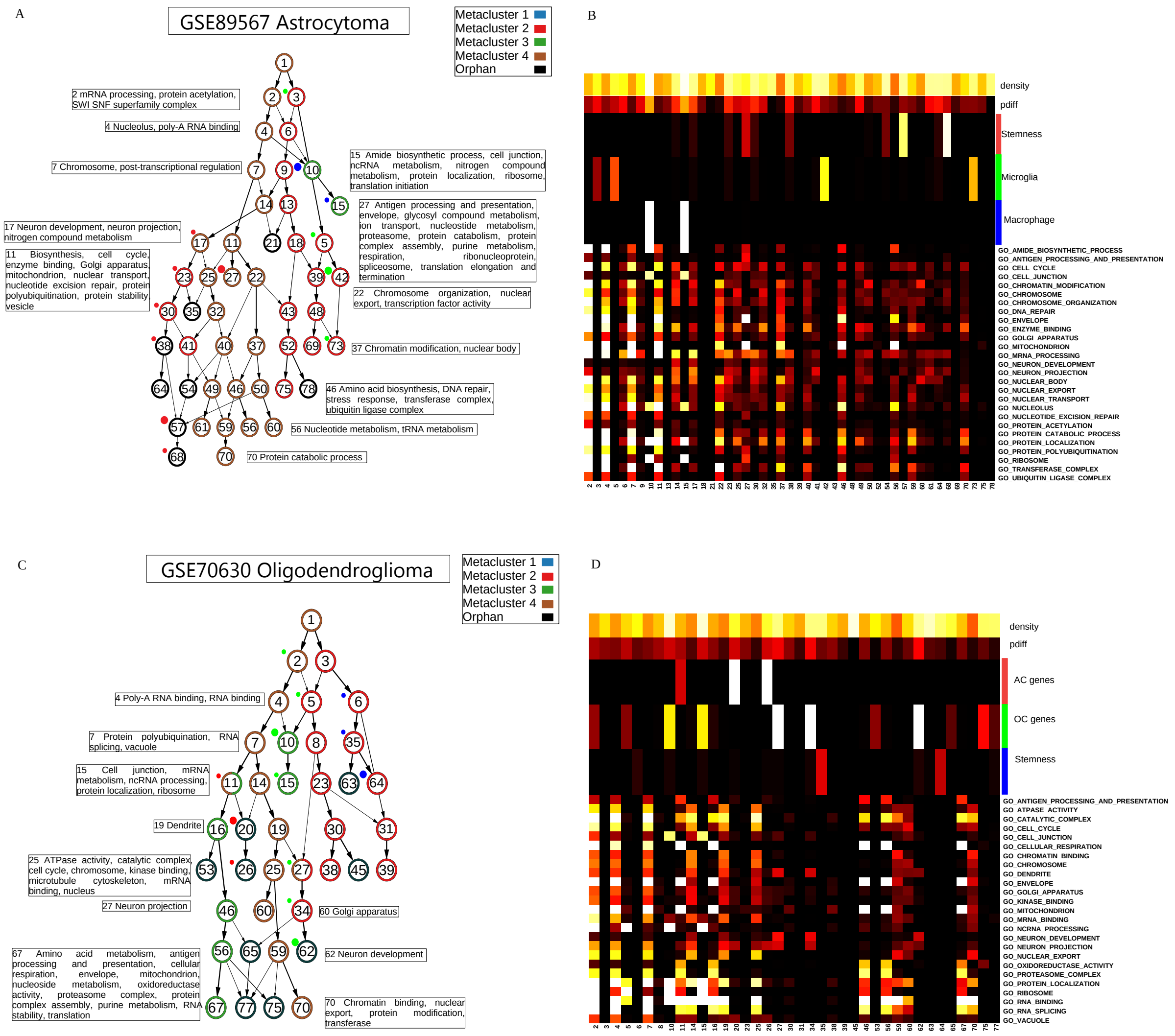

Fig. S8. Summary information of clustering results of GSE89567 astrocytoma and GSE70630 oligodendroglioma data. Legend follows Figure 2.

Biology Open • Supplementary information

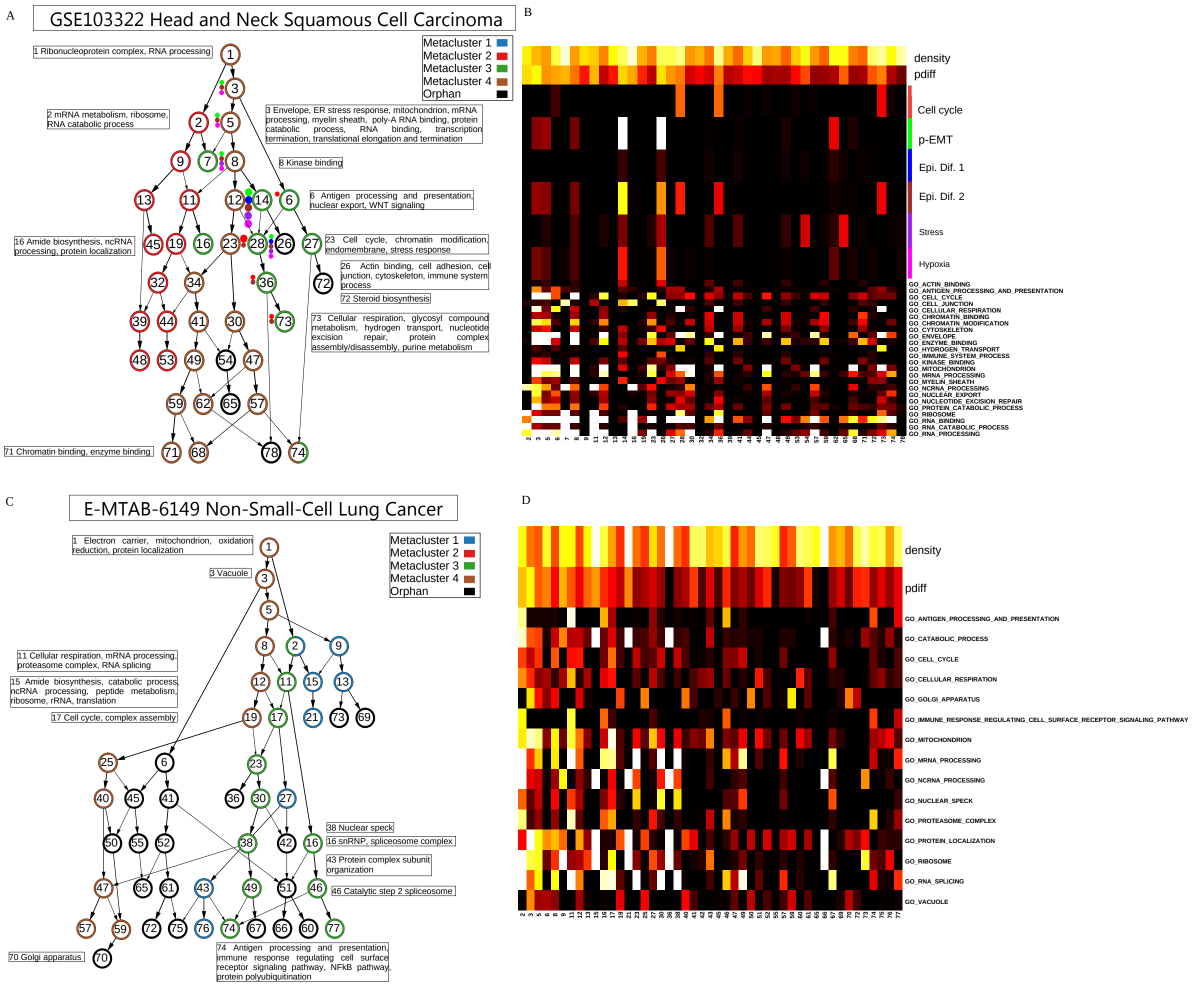

Fig. S9. Summary information of clustering results of GSE103322 head and neck squamous cell carcinoma and E-MTAB-6149 non-small-cell lung carcinoma data. Legend follows Figure 2.

Biology Open • Supplementary information

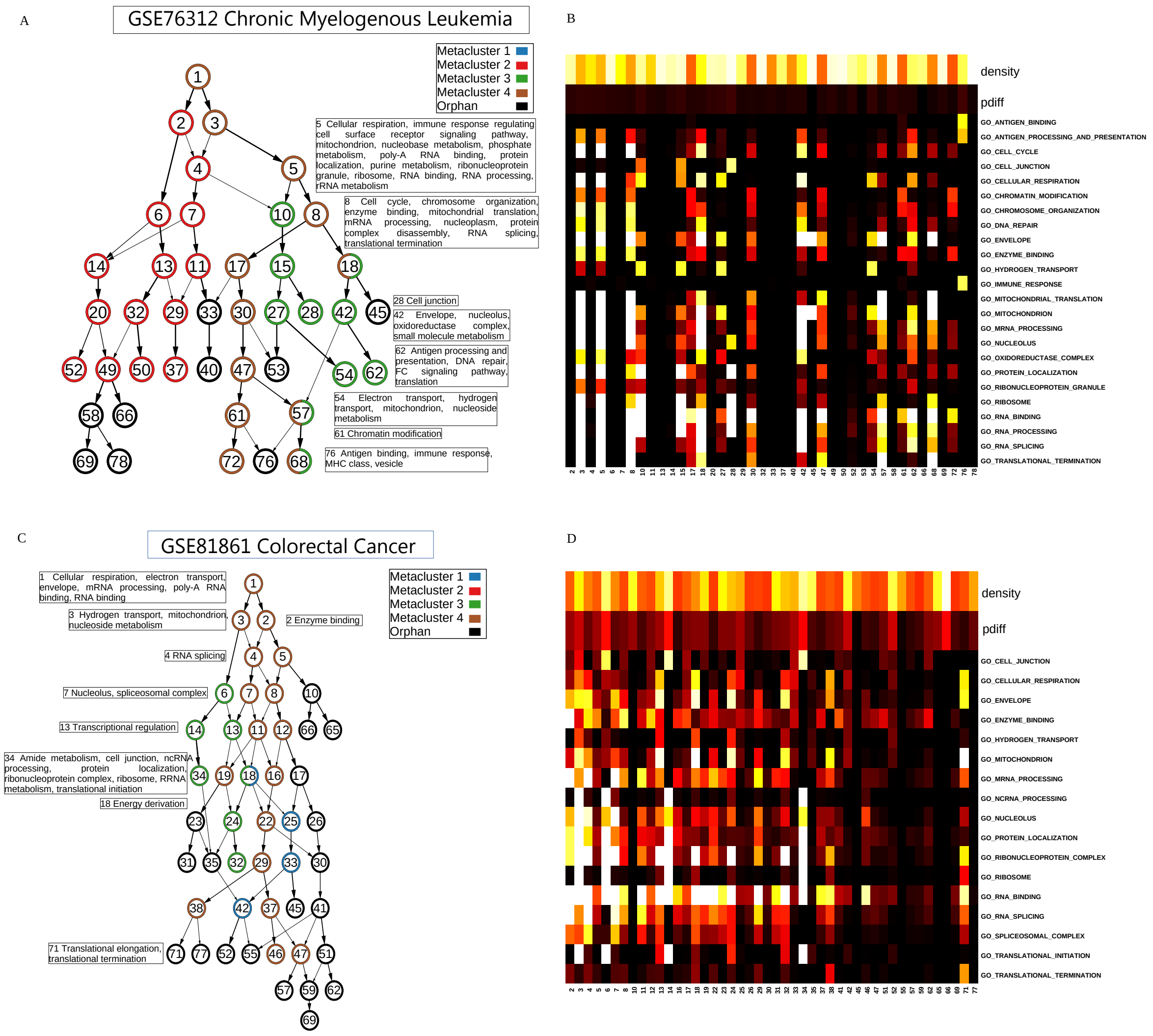

Fig. S10. Summary information of clustering results of GSE76312 chronic myeloid leukemia and GSE81861 colorectal cancer data. Legend follows Figure 2.

Biology Open • Supplementary information

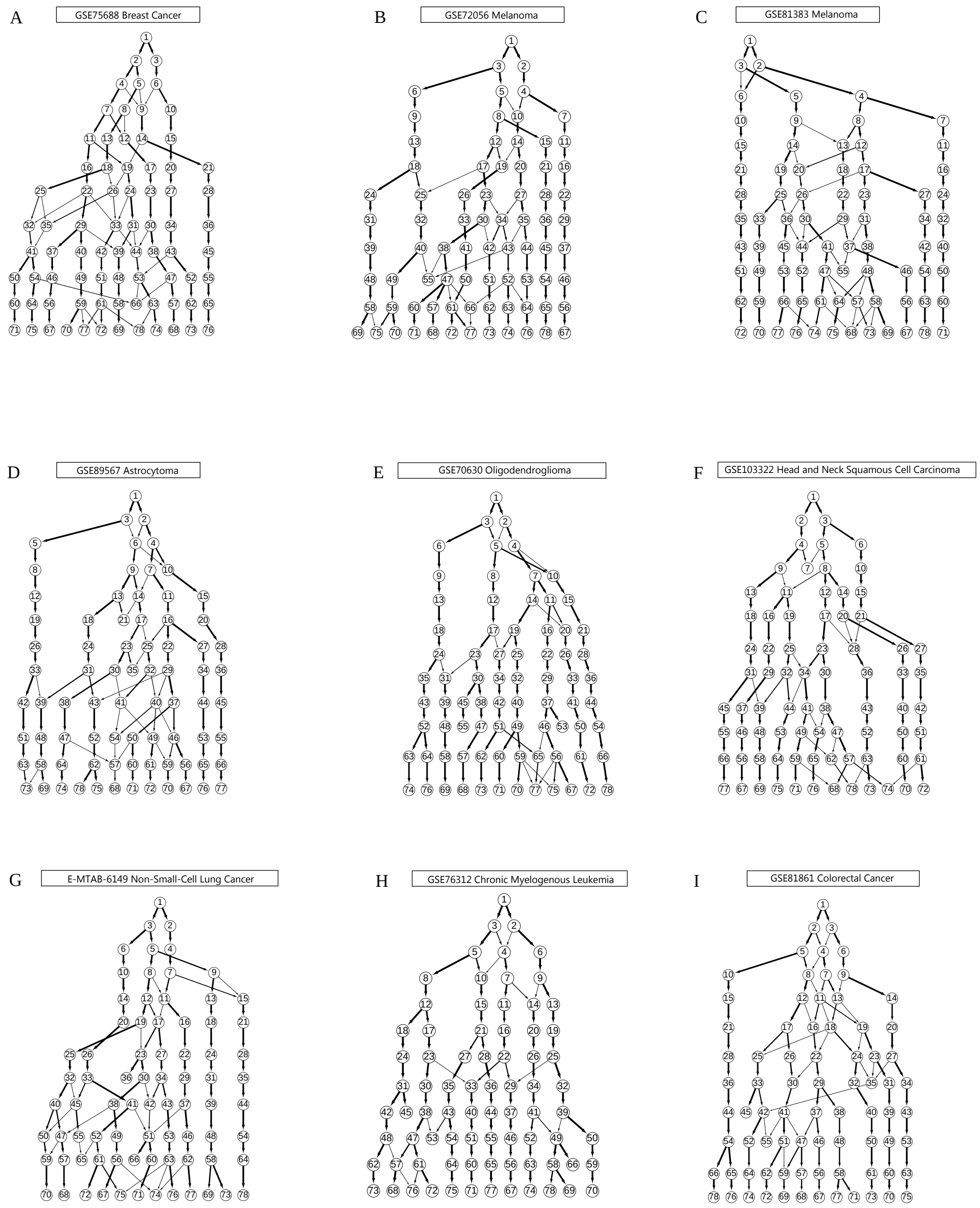

Fig. S11. The full hierarchies of k-means clusters ($k = 1 - 12$) of the nine sc-RNAseq datasets.

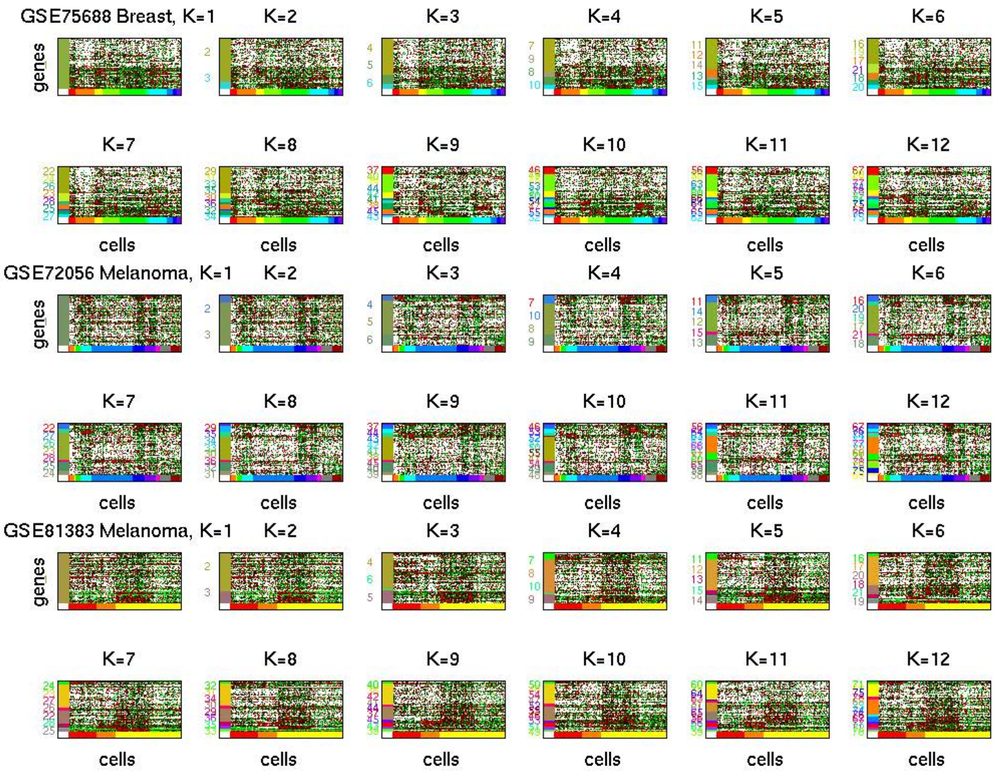

Fig. S12. Expressions of gene clusters of GSE75688 breast cancer data, GSE72056 melanoma data, and GSE81383 melanoma data from $k = 1$ to 12. In each panel, rows are genes sorted by their cluster identities and columns are cancer cells sorted by their tumor identities. Color annotation of a gene cluster at level k is a mixture of color annotations of parent gene clusters at level $k - 1$.

Biology Open • Supplementary information

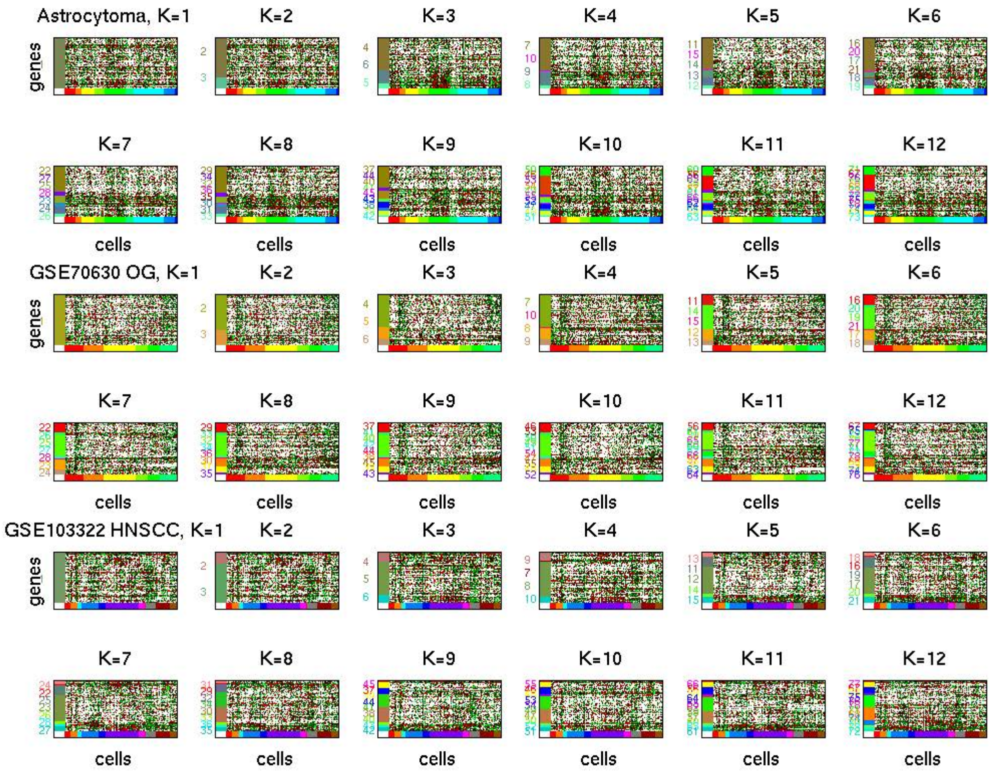

Fig. S13. Expressions of gene clusters of GSE89567 astrocytoma data, GSE70630 oligodendroglioma data, and GSE103322 head and neck squamous cell carcinoma data from $k = 1$ to 12. Legend follows Figure S12.

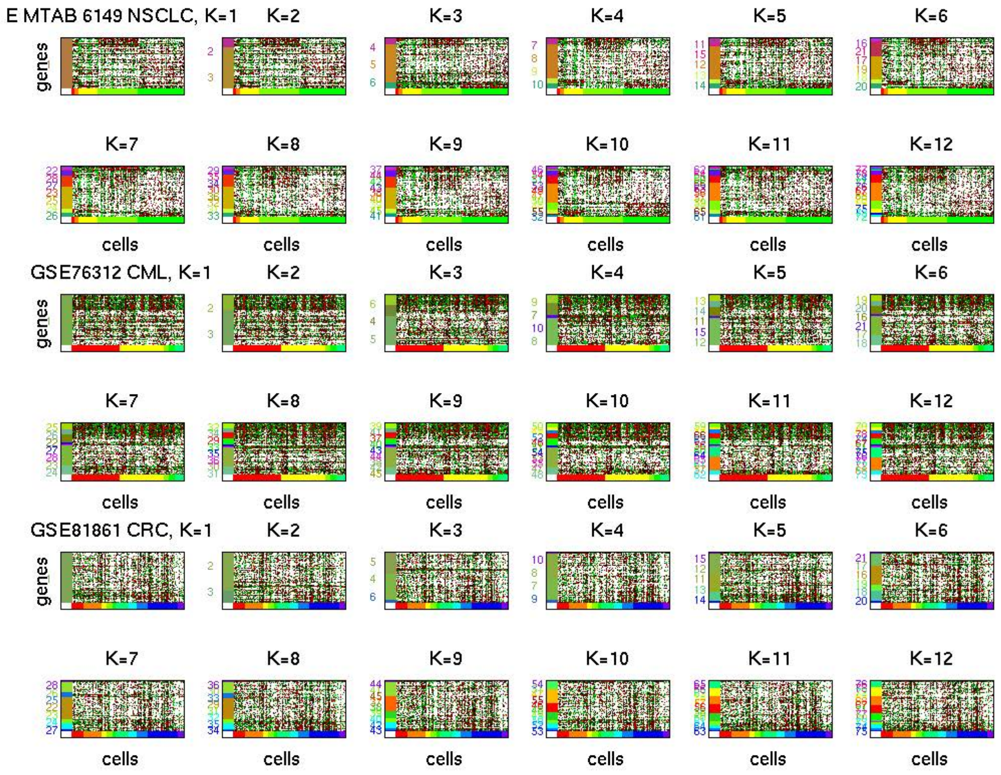

Fig. S14. Expressions of gene clusters of E-MTAB-6149 non-small cell lung cancer data, GSE76312 chronic myeloid leukemia data, and GSE81861 colorectal cancer data from $k = 1$ to 12. Legend follows Figure S12.

Biology Open • Supplementary information

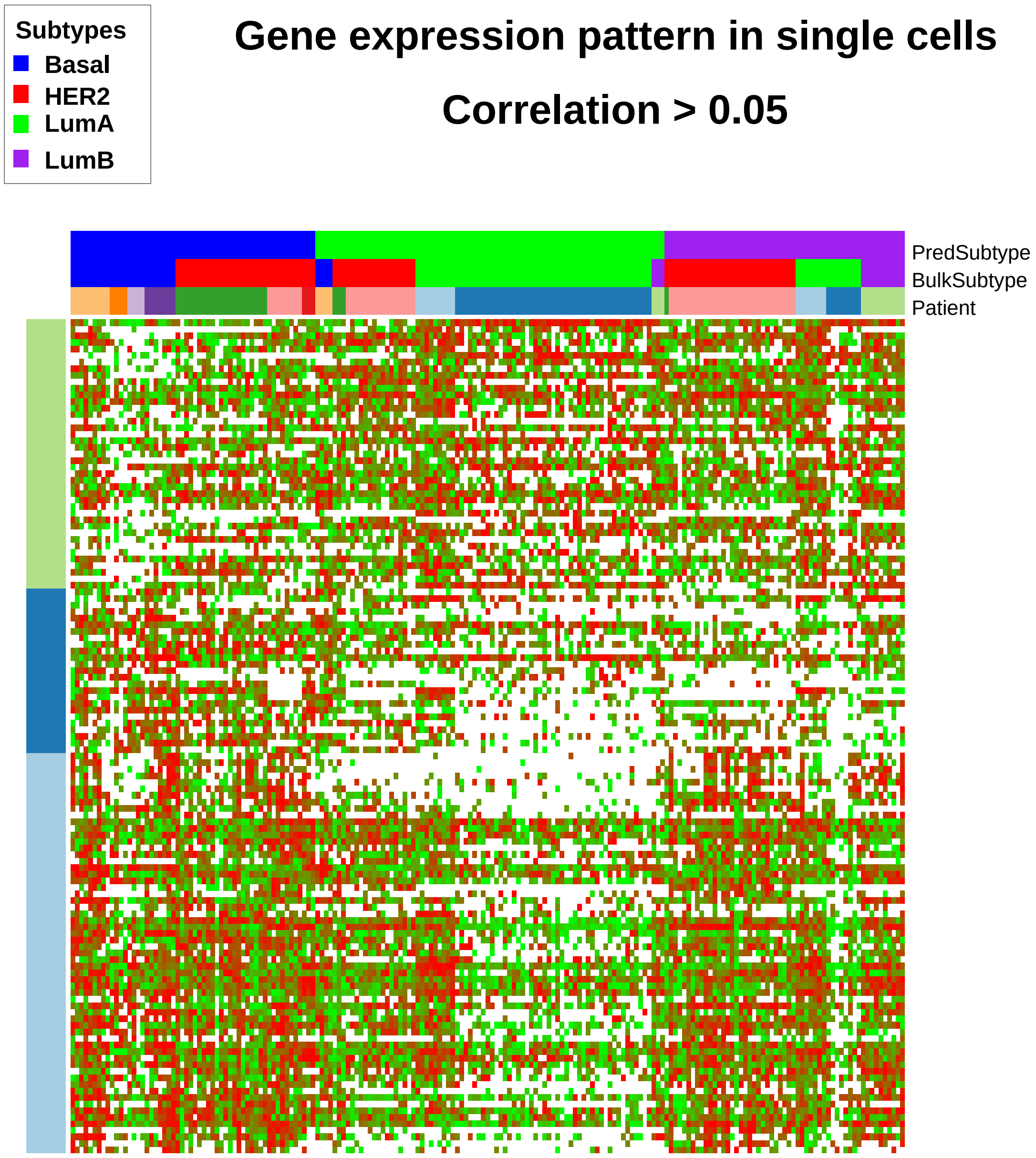

Fig. S15. The combinatorial expression patterns of extended PAM50 genes of breast cancer sc-RNAseq data among the cells with the correlation coefficients with the bulk data > 0.05. The organization of genes and cells follow Figure 4D and 4E.

Biology Open • Supplementary information

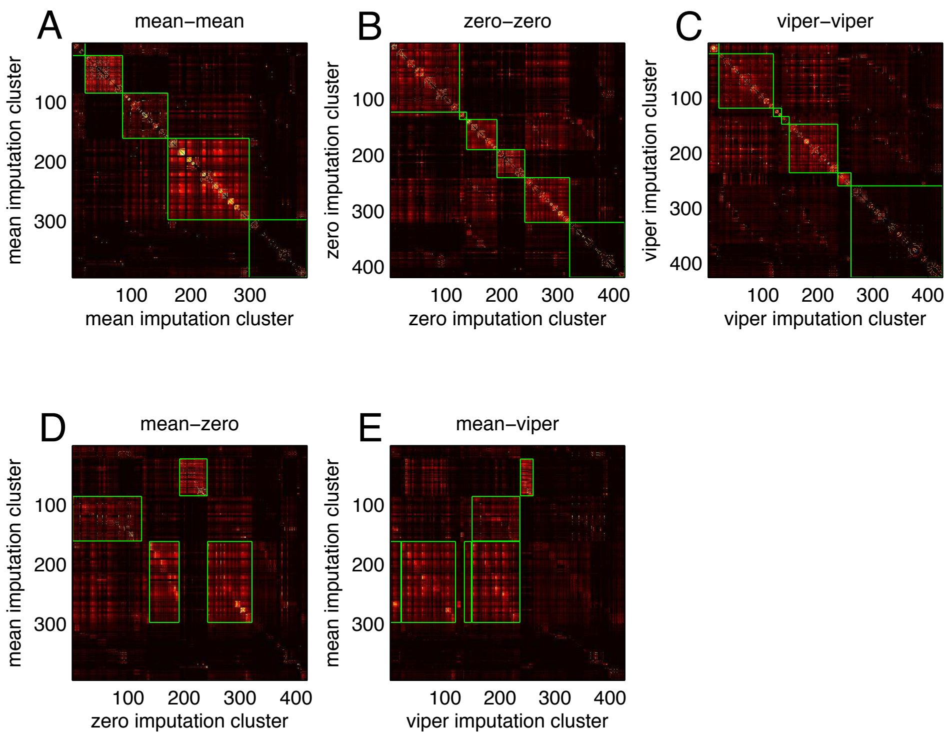

Fig. S16. The overlap rates between gene clusters (Jaccard indices) sorted by meta gene cluster identities. Panels A-C display the overlap rates within gene clusters using mean, zero and VIPER imputations respectively. Panels D-E display the overlap rates of gene clusters between mean and zero imputations (D) and between mean and VIPER imputations (E). Within each panel, clusters belonging to the same meta gene clusters are marked by green boxes. In panels A-C the lower-right boxes collect all orphan clusters for each imputation method. In panels D-E green boxes mark the meta gene cluster pairs with moderate overlap rates.

Biology Open • Supplementary information

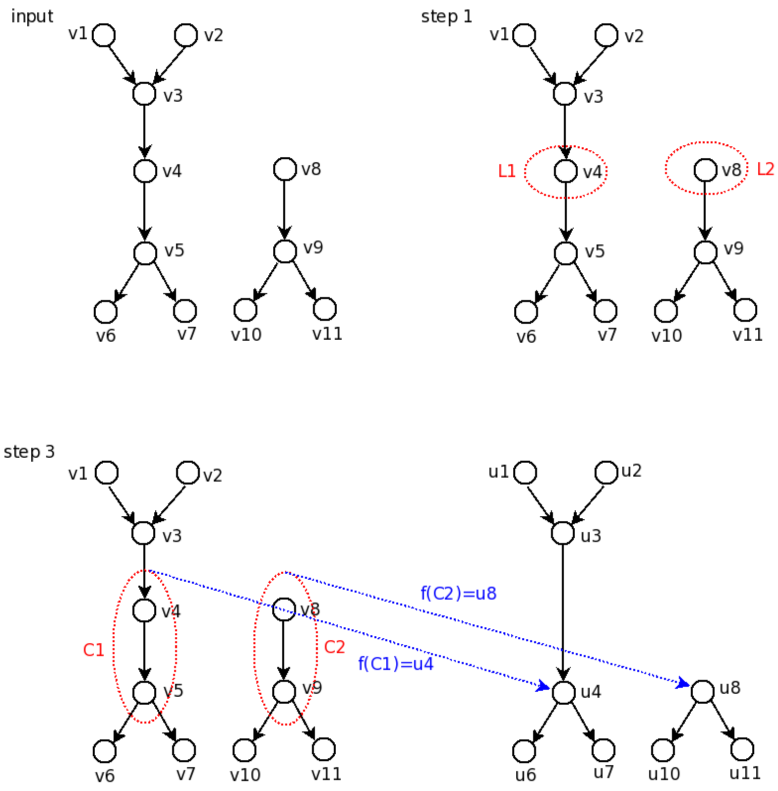

Fig. S17. A toy example illustrating the cluster hierarchy reduction algorithm. The inputs are two directed acyclic graphs. Subtrees without branches (stumps) are collapsed into single nodes.

Biology Open • Supplementary information

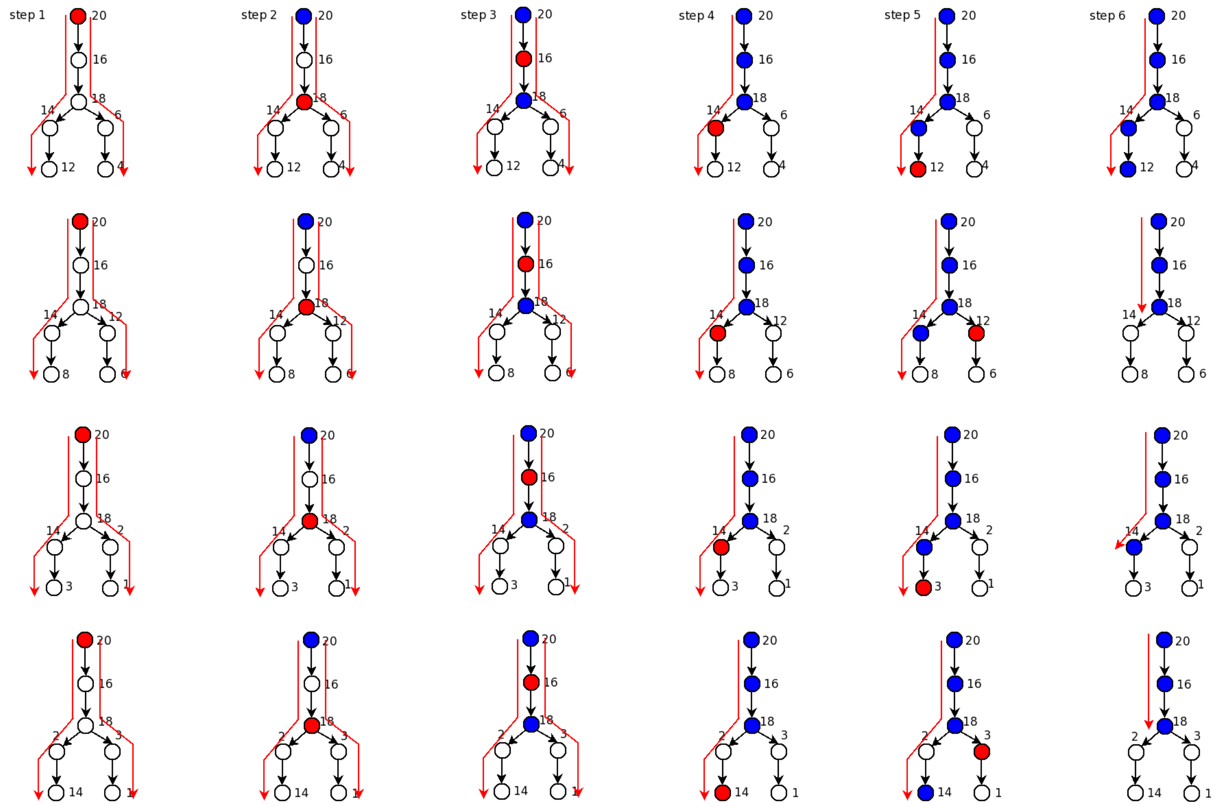

Fig. S18. Four toy examples illustrating the unique enrichment determination algorithm. Each row denotes an example, and each column denotes a step when running the algorithm. Numbers denote the transformed scores of the clusters. Red nodes denote the sorted clusters examined at the current step. Blue nodes denote the sorted clusters in the list L. Red paths denote the candidate paths P.

Biology Open • Supplementary information

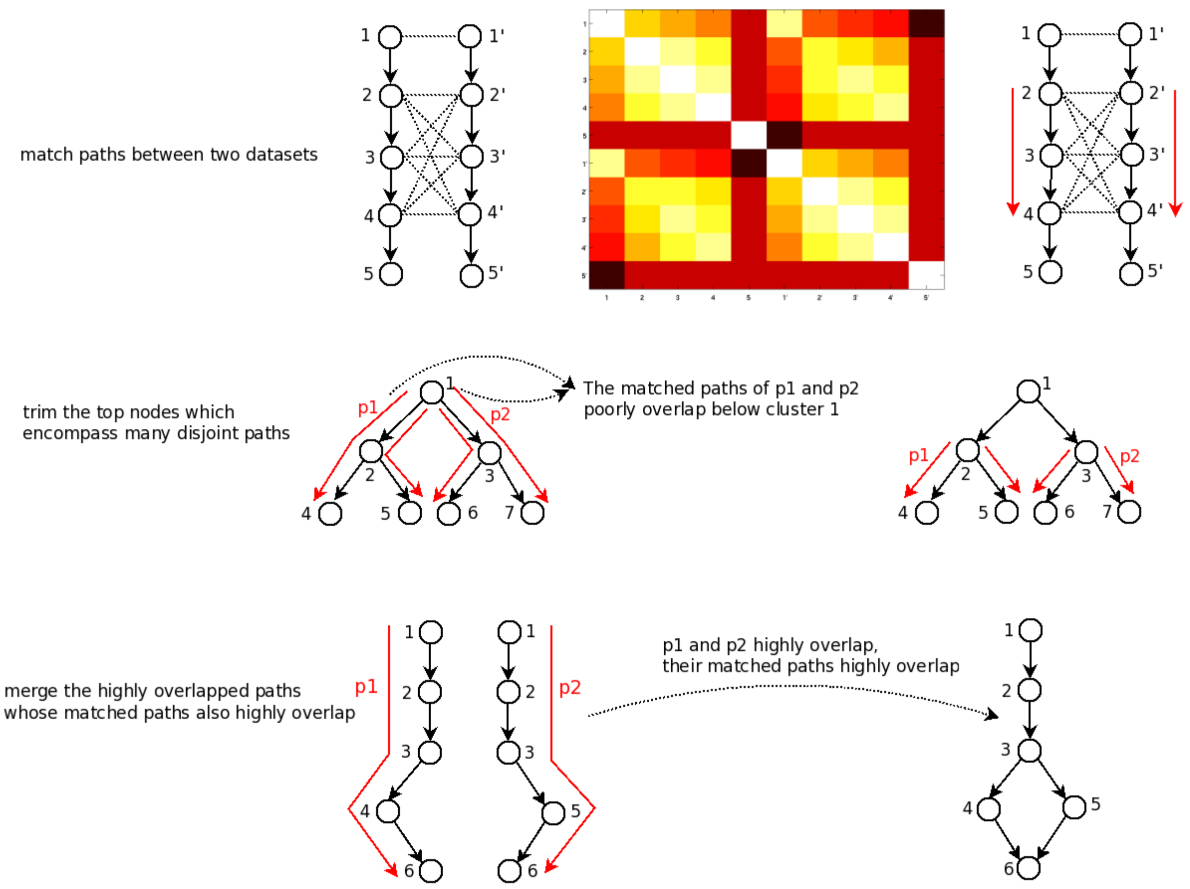

Fig. S19. Toy examples illustrating part 1 of the gene cluster hierarchy alignment algorithm. In the top row, two paths from the two datasets ($1 \to 2 \to 3 \to 4 \to 5$ and $1' \to 2' \to 3' \to 4' \to 5'$) are compared, and the maximally matched subpaths $2 \to 3 \to 4$ and $2' \to 3' \to 4'$ are identified. In the middle row, the top node 1 for four paths is removed as it encompasses disjoint downstream subpaths which are also poorly overlapped in the matched paths in other datasets. In the bottom row, two paths p_1 and p_2 highly overlap thus are merged to form a subgraph.

Biology Open • Supplementary information

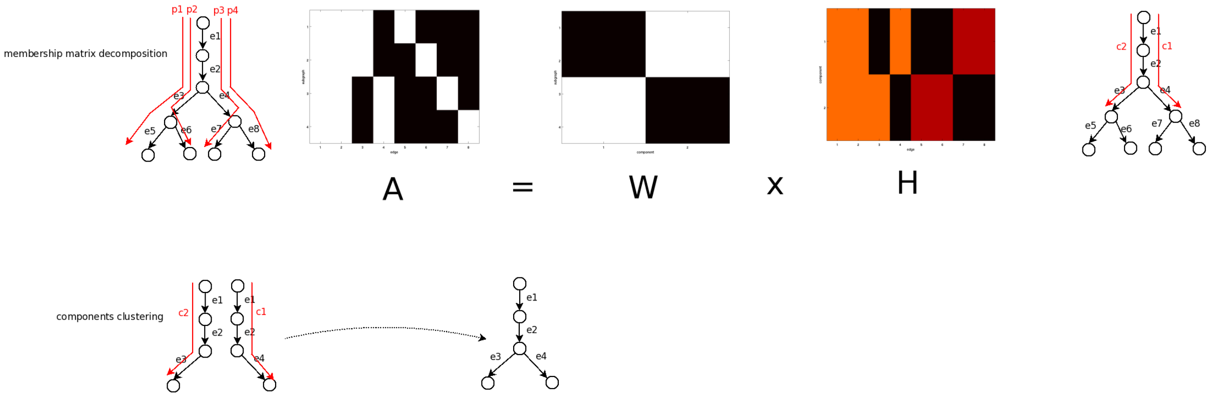

Fig. S20. Toy examples illustrating part 2 of the gene cluster hierarchy alignment algorithm. In the top row, the membership matrix A of 4 subgraphs p_1, p_2, p_3, p_4 over 8 edges is decomposed by NMF into two components e_1, e_2, e_4 and e_1, e_2, e_3 in H. In the bottom row, the two components are merged to form a meta cluster.

Biology Open • Supplementary information

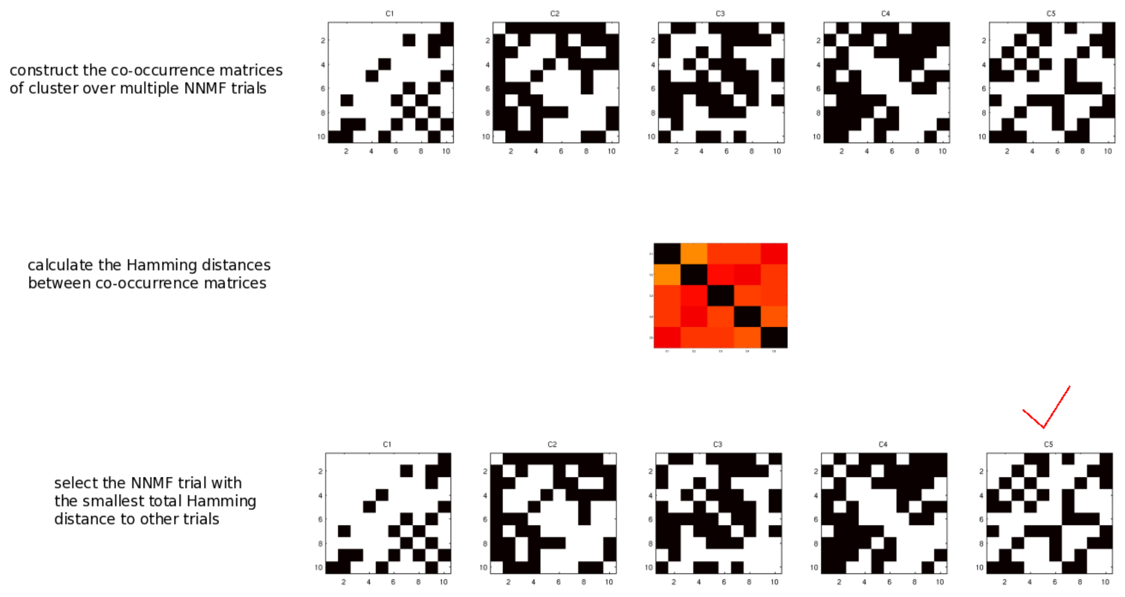

Fig. S21. Toy examples illustrating part 3 of the gene cluster hierarchy alignment algorithm. The top and bottom rows display the cluster co-occurrence matrices of meta clusters from 5 NMF trials. The middle row displays the pairwise Hamming distances of the co-occurrence matrices. Trial 5 is selected since it has the smallest total Hamming distance relative to all other trials.

Table S1. Three indices of transcriptional heterogeneity/homogeneity – average NSV fraction, p_{dff} score, and average Shannon's entropy – in three cases of simulated data with increasing levels of heterogeneity and five noise levels (standard deviation).

Click here to download Table S1

Table S2. Enrichment outcomes of MSigDB gene sets in seven clusters for each dataset. Table S2A: FDR-adjusted enrichment p-values of type 1 and type 2 gene sets. Table S2B: Summary of enriched functional categories in each gene cluster.

Click here to download Table S2

Table S3. Full gene cluster members of the nine datasets.

Click here to download Table S3

Table S4. Reduced gene cluster members of the nine datasets.

Click here to download Table S4

Table S5. The mapping from full gene cluster indices to reduced gene cluster indices of the nine datasets.

Click here to download Table S5

Table S6. The enrichment FDR-adjusted p-values of selected GO terms in full gene clusters of the nine datasets.

Click here to download Table S6

Table S7. The enrichment FDR-adjusted p-values of selected GO terms in reduced gene clusters of the nine datasets.

Click here to download Table S7

Table S8. The uniquely enriched GO terms and (FDR-adjusted p-values) in full gene clusters of the nine datasets.

Click here to download Table S8

Table S9. The hyper-geometric enrichment p-values of marker genes reported in the datasets in full gene clusters (S9A) and reduced gene clusters (S9B).

Click here to download Table S9

Table S10. The NSV and scores and densities of all full gene clusters.

Click here to download Table S10

Table S11. Assignments of gene clusters to meta gene clusters.

Click here to download Table S11

Table S12. GO term enrichment FDR-adjusted p-values of meta gene clusters.

Click here to download Table S12

Table S13. The number of orphan clusters with significant enrichment in selected GO terms and each dataset.

Click here to download Table S13

Table S14. The enrichment outcomes of selected GO terms and breast cancer single-cell gene clusters in each PAM50 gene group.

Click here to download Table S14

Table S15. Characteristics of gene clusters with k k k for dataset by employing three imputation criteria: mean (sheet A), zero (sheet B), and VIPER (sheet C). In each sheet, we report the k_{kkkk} scores and densities of valid entries, and enrichment outcomes of four functional categories – cell cycle, ribosome, respiration, and RNA splicing. ++ and + denote strong (p-value $k\ kk^{kkk}$) and moderate (p-value $k\ kk^{kkk}$ and $k\ kk^{kk}$) enrichment respectively.

Click here to download Table S15
